# A near-global, high resolution land surface parameter dataset for the variable infiltration capacity model

**DOI:** 10.1038/s41597-021-00999-4

**Published:** 2021-08-11

**Authors:** Jacob R. Schaperow, Dongyue Li, Steven A. Margulis, Dennis P. Lettenmaier

**Affiliations:** 1 Department of Civil and Environmental Engineering, University of California, Los Angeles, 90095 USA; 2 Department of Geography, University of California, Los Angeles, 90095 USA

**Keywords:** Hydrology, Hydrology, Water resources, Climate and Earth system modelling

## Abstract

Hydrologic models predict the spatial and temporal distribution of water and energy at the land surface. Currently, parameter availability limits global-scale hydrologic modelling to very coarse resolution, hindering researchers from resolving fine-scale variability. With the aim of addressing this problem, we present a set of globally consistent soil and vegetation parameters for the Variable Infiltration Capacity (VIC) model at 1/16° resolution (approximately 6 km at the equator), with spatial coverage from 60°S to 85°N. Soil parameters derived from interpolated soil profiles and vegetation parameters estimated from space-based MODIS measurements have been compiled into input files for both the Classic and Image drivers of the VIC model, version 5. Geographical subsetting codes are provided, as well. Our dataset provides all necessary land surface parameters to run the VIC model at regional to global scale. We evaluate VICGlobal’s ability to simulate the water balance in the Upper Colorado River basin and 12 smaller basins in the CONUS, and their ability to simulate the radiation budget at six SURFRAD stations in the CONUS.

## Abstract (summary)

**Table:** 

Measurement(s)	vegetation characteristics • soil characteristics • elevation
Technology Type(s)	satellite imaging • soil sampling • digital curation
Factor Type(s)	geographic location
Sample Characteristic - Environment	vegetation layer • soil
Sample Characteristic - Location	global

Machine-accessible metadata file describing the reported data: 10.6084/m9.figshare.14869500

## Background & Summary

The Variable Infiltration Capacity (VIC, https://github.com/UW-Hydro/VIC) model is a macroscale, semi-distributed hydrologic model^1–3^ that calculates land surface states and fluxes by solving the surface water and energy balances. The model has a wide user base — the citation index Web of Science shows the original VIC paper^3^ has been cited nearly 2000 times, with contributing authors from at least 56 countries. Despite the model′s popularity, there are only a few ready-made soil and vegetation parameter datasets that modelers can use to run VIC outside the continental United States. Previous global input datasets^4–8^ have been compiled for VIC at resolutions ranging from 2° to 1/4°. Many studies, including Su *et al.*^5^, Zhou *et al.*^7^, and Adam *et al.*^8^ use parameters based on the 2° soil and vegetation parameters developed by Nijssen *et al.*^4^ (henceforth N2001). As useful as the N2001 dataset has been over the years, the VIC-modeling community would be well-served by a higher-resolution update. The N2001 dataset and its derivatives are limited by the dataset’s coarse resolution, geographically sparse subset of leaf-area index observations, and assumptions of temporally-invariant albedo and 100 percent canopy coverage for all land cover classes, as noted by Bohn and Vivoni^9^, who developed a new VIC parameter dataset for North America that addresses these issues. Our dataset, VICGlobal, emulates their approach at a global-scale.

VICGlobal’s predecessor, the N2001 soil and vegetation parameters, may be appropriate for continental-scale modelling, but its coarse resolution makes it less useful for parameterizing VIC at smaller scales. Coarse resolution land surface models miss topographic variability, distort river networks, and prevent proper representation of land-atmosphere interactions in coupled land-atmosphere models. At coarse resolution, topographic characteristics such as elevation and vegetation cover are averaged over a large grid cell, so the model will miss key details such as the effect of terrain on the radiation balance and the effect of vegetation on ET-soil moisture partitioning. This is particularly important in mountainous regions, where there are large changes in topography across relatively small areas. While VIC does not represent fluxes from one grid cell to another, it is frequently coupled to a routing model to simulate how runoff flows between grid cells. At very coarse resolutions, the modelled river network loses its resemblance to the true river network, necessitating upscaling algorithms to obtain usable coarse-resolution river networks (e.g. Wu *et al.*^10^). Finally, high resolution land surface modelling could improve our ability to simulate land-atmosphere interactions that occur over relatively small spatial scales^11^. With 1/16° grid cells and representation of up to 17 land cover classes within each grid cell, VICGlobal is a step toward addressing each of these resolution-related challenges.

Regional-scale VIC inputs at 1/16° resolution already exist but have limited coverage outside North America. Livneh *et al.*^12^ (henceforth L2013) set up the VIC model over the conterminous United States (CONUS) using soil and vegetation data compiled from sources including the Food and Agriculture Organization (FAO)/UNESCO Soil Map of the World and the Advanced Very High Resolution Radiometer (AVHRR). The L2013 VIC parameterization is based on that of Maurer *et al.*^13^, with calibration for a better match with streamflow data. Bohn and Vivoni^9^ (henceforth BV2019) released an updated 1/16° vegetation parameter dataset for the CONUS, Mexico, and part of Canada, improving on of the limitations of the L2013 dataset, such as its assumption of temporally-invariant albedo. They estimated time-varying albedo, leaf-area index (LAI), and fractional canopy cover using observations from the Moderate Resolution Imaging Spectroradiometer (MODIS).

Drawing on the L2013 and BV2019 VIC parameterizations, we developed VICGlobal, a near-global dataset of soil and vegetation parameters for the VIC model at 1/16° resolution, which VIC users can download and subset to their region of study. We estimated soil parameters based on the 30 arc-second FAO Harmonized World Soil Database^14^ (HWSD). Vegetation parameters are based on 500 m resolution MODIS observations. VICGlobal includes all the necessary parameters to run regional- to global-scale VIC simulations. We provide MATLAB® codes to subset the VICGlobal parameters to a particular domain. In addition to parameters, meteorological forcing data are required to run VIC. We do not include meteorological forcing data as part of VICGlobal. Instead, we direct readers to existing forcing datasets with near-global coverage, such as the reanalysis datasets MERRA-2^15^ and GLDAS^16^, or real-time measurement-based datasets — see e.g. Xiao *et al.*^17^, Livneh *et al.*^12,18^, Bohn *et al.*^19^

Finally, a note on the file format: The upgrade from VIC version 4 (VIC-4) to VIC version 5 (VIC-5) introduced two “drivers” for running the model. The Image driver takes NetCDF files as inputs, while the Classic driver takes ASCII text files. The VICGlobal parameter files are available in two formats: one for VIC-5 Classic, and one for VIC-5 Image.

## Methods

This section describes how we used freely-available data to compile Classic driver input files for the VIC model. First, we created parameter files for VIC-5 Classic, then we converted them to NetCDF format for VIC-5 Image. VIC-5 Classic requires three parameter files: a soil parameter file, a vegetation parameter file, and a vegetation library file. An optional elevation band file can be provided to resolve sub-grid variability in elevation, which is important in regions with complex topography. The parameters are arranged as a relational database: each grid cell has a unique identifier, called a grid cell number, in the soil parameter file, that VIC uses to find the corresponding rows of data in the vegetation parameter and elevation band files. The Image driver uses a different setup, with all parameters stored in a single NetCDF file.

### Soil parameters

The soil parameter file for VIC-5 Classic is an ASCII text file that includes soil parameters such as hydraulic conductivity and porosity, but also other kinds of static parameters, such as average precipitation and time zone offset from GMT. Each row of the soil parameter file represents one grid cell, and each column represents a different variable. We compiled the soil parameter file using MERIT^20^ elevation data, soil texture data from the FAO HWSD, pedotransfer tables relating soil texture to other soil properties, and interpolated weather station data (WorldClim^21^). Any remaining parameters were set to suggested values from the VIC model’s documentation^2^. The following sections describe the estimation of each variable in the soil parameter file, summarized in Table 1.

**Table 1:** VIC model parameters for the soil parameter file.

Soil parameter	Description	Source
*run_cell*	Flag for running this cell	
*grid_cell*	Grid cell number	
*lat*	Latitude	MERIT^20^
*lon*	Longitude	MERIT
*b*_*infilt*_	Variable infiltration capacity parameter	VIC documentation^2^
*ds*	Fraction of *Dsmax* where nonlinear baseflow occurs	VIC documentation^2^
*dsmax*	Maximum velocity of baseflow (mm/day)	HWSD*
*ws*	Fraction of maximum soil moisture where nonlinear baseflow occurs	VIC documentation^2^
*c*	Exponent used in baseflow curve	VIC documentation^2^
***expt***	Exponent in Campbell’s equation for hydraulic conductivity	HWSD*
***K***_***sat***_	Saturated hydraulic conductivity (mm/day)	HWSD*
***phi***_***s***_	Soil moisture diffusion parameter in each soil layer (not used)	Not used
***initm***	Initial moisture content (mm)	HWSD*
*elev*	Elevation (m)	MERIT
***depth***	Thickness of each soil layer (m)	HWSD^14^
*avg_T*	Average soil temperature (°C)	WorldClim^21^
*dp*	Soil thermal damping depth (m)	VIC documentation^2^
***bubble***	Bubbling pressure of soil (cm)	HWSD*
***quartz***	Quartz content	HWSD*
***bulk_density***	Bulk density of soil (kg/m^3^)	HWSD^14^
***soil_density***	Soil particle density (kg/m^3^)	VIC documentation^2^
*offgmt*	Time zone offset relative to GMT (hr)	Based on longitude
***wcr***_***fract***_	Fractional soil moisture content at critical point	HWSD*
***wpwp***_***fract***_	Fractional soil moisture content at wilting point	HWSD*
*Rough*	Surface roughness length of bare soil (m)	VIC documentation^2^
*snow_rough*	Surface roughness length of snowpack (m)	VIC documentation^2^
*annual_prec*	Average annual precipitation (mm)	WorldClim^21^
***resid_moist***	Residual moisture fraction	Assumed zero
*fs_active*	Flag for whether to run frozen soils module	
*July_Tavg*	Average July temperature (°C)	WorldClim^21^

Parameters whose source is “VIC documentation” were set to suggested values from the VIC documentation^2^. Bold parameters have distinct values in each of the three soil layers.

^*^Calculated based on HWSD soil texture data, using pedotransfer table (Table 2).

### Elevation and land mask

The VICGlobal soil parameter file uses the Multi-Error-Removed Improved-Terrain (MERIT^20^) digital elevation model (DEM) to define the elevations, latitudes, and longitudes of each land grid cell. The MERIT DEM is an error-corrected and extended version of the SRTM DEM, with 3 arc-second resolution and coverage from 60°S to 85°N and 180°W to 180°E. Specifically, MERIT is a combination of the SRTM, AW3D, and Viewfinder Panoramas’ DEMs, corrected for striping, speckle, absolute bias, and tree height bias. We used bilinear interpolation to aggregate MERIT to 1/16° resolution and derive a 1/16° MERIT-based land mask and DEM (Figure S1).

### Soil texture data

**Fig. 1:**
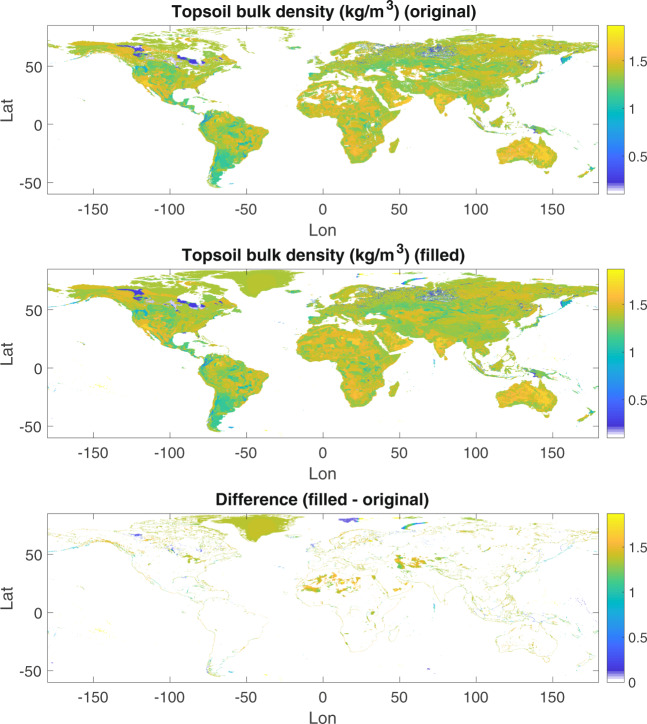
Bulk density data from the Harmonized World Soils Database (HWSD). The top panel shows HWSD bulk density data resampled to 1/16° resolution, the middle panel shows bulk density after infilling holes, and the bottom panel shows the difference.

Soil texture (percent sand, silt, and clay) and bulk density were obtained from the FAO HWSD, a gridded soil parameter dataset derived from *in-situ* measurements of the soil column. We used a 0.05° resolution NetCDF dataset converted from the original HWSD Microsoft Access database by Wieder *et al.*^22^. We resampled the HWSD soil data from 0.05° to 1/16° resolution using bilinear interpolation with the MATLAB® function *griddedInterpolant*. While HWSD has near global coverage, there are missing data in some places around the world, notably Greenland and northern Africa. We filled in these missing data using inpainting, a gap-filling method from the field of image processing. We used the MATLAB® function *inpaintnans*^23^, which uses a partial differential equation method to fill in missing data, to fill gaps in the HWSD data over the MERIT land mask. Figure 1 shows the HWSD bulk density data before and after inpainting.

The HWSD data are divided into “topsoil” and “subsoil” parameters. The first 30 cm of the soil column are considered topsoil and the lower 70 cm subsoil. VIC is typically run with three soil layers, so we created a three-layer soil parameter file by breaking up the 30 cm HWSD topsoil layer into two soil layers: one of 10 cm and one of 20 cm, so the final soil parameter file has three layers, with thicknesses of 10 cm, 20 cm and 70 cm, from top to bottom of the soil column. Ten centimeters has been a common choice for the uppermost layer soil depth in VIC modeling applications since its use by Liang *et al.*^24^. Soil layer depths are typically used as calibration parameters. VICGlobal values should be considered a starting estimate.

### Calculating soil parameter values based on soil textures

**Fig. 2:**
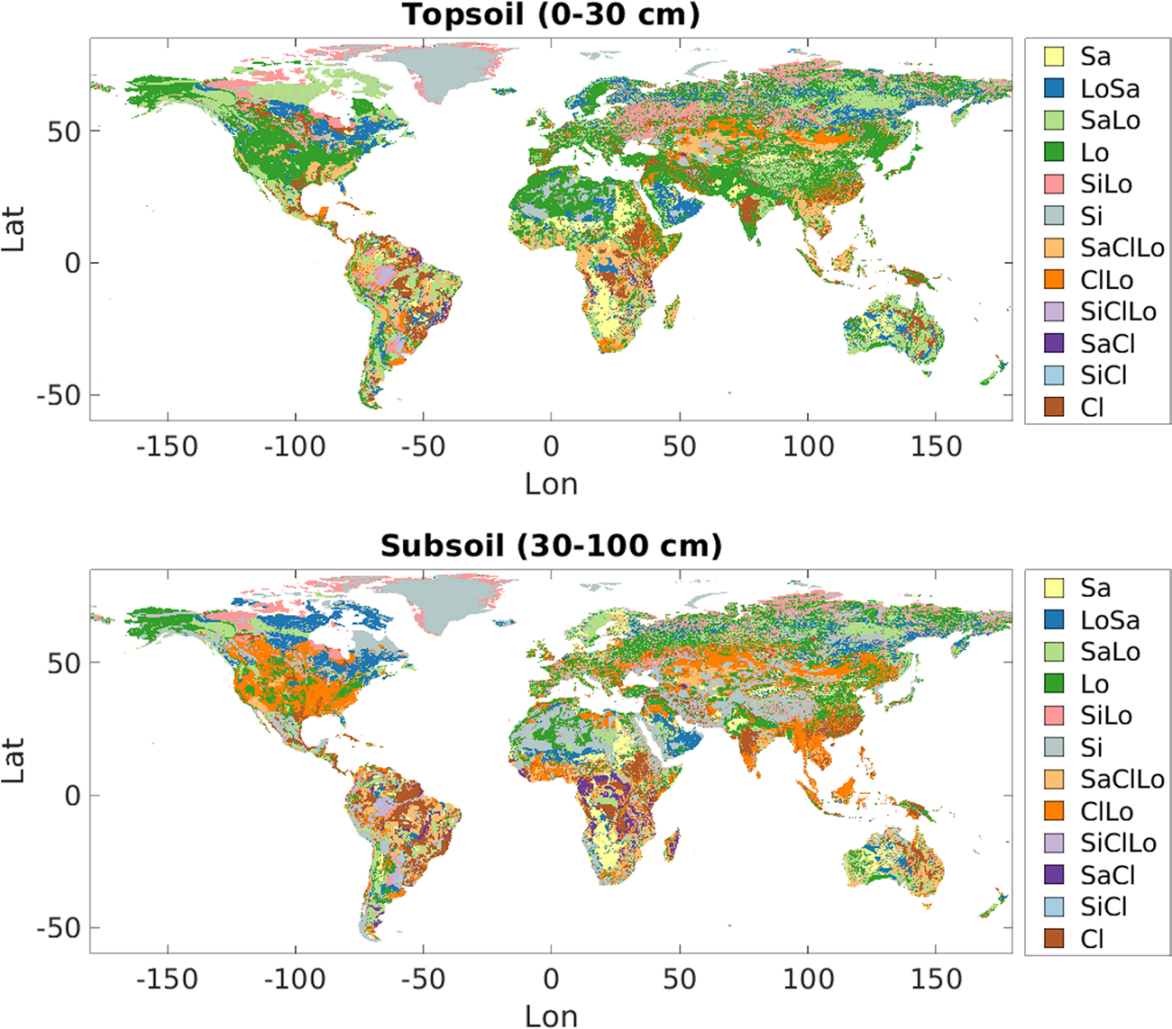
USDA soil texture classifications based on HWSD. Topsoil is soil from 0–30 cm below the surface, and subsoil is soil between 30–100 cm deep.

Pedotransfer functions (e.g. Cosby *et al.*^25^) relate readily available soil properties, such as soil texture, to less easily-observed properties, such as hydraulic conductivity. After resampling the HWSD data from 1/4° to 1/16° resolution, we estimated soil parameters by classifying each grid cell’s USDA soil texture class and assigning physical soil properties based on a lookup table included with the VIC documentation^2,26^. The lookup table (Table 2) relates the 12 USDA soil texture classes to bulk density, field capacity, wilting point, porosity, saturated hydraulic conductivity, and slope of the soil water retention curve in Campbell’s equation. We classified soil textures using the USDA soil texture triangle, as implemented by the MATLAB® function *soil_classification*^27^. Figure 2 shows the derived USDA soil texture map. We used these along with the lookup table to estimate saturated hydraulic conductivity (*K*_*sat*_), the exponent in Campbell’s equation for hydraulic conductivity (*expt*), fractional soil moisture at the critical point (*wcr*_*fract*_), where the critical point is about 70% of field capacity, fractional soil moisture at the wilting point (*wpwp*_*fract*_), quartz content, and porosity for each soil layer. The lookup table^26^ did not include quartz content, so we supplemented it with the soil texture-quartz content lookup table from Peters-Lidard *et al.*^28^.

**Table 2:** USDA soil texture class lookup table.

USDA class	Field capacity	Wilting point	Porosity	*K*_*sat*_ (cm/hr)	Slope (*b*) of the retention curve, in log space	Quartz content
Sand	0.08	0.03	0.43	38.41	4.1	0.95
Loamy sand	0.15	0.06	0.42	10.87	3.99	0.85
Sandy loam	0.21	0.09	0.40	5.24	4.84	0.69
Loam	0.32	0.12	0.46	3.96	3.79	0.19
Silt loam	0.28	0.08	0.52	8.59	3.05	0.05
Silt	0.29	0.14	0.43	1.97	5.30	0.41
Sandy clay loam	0.27	0.17	0.39	2.40	8.66	0.61
Clay loam	0.36	0.21	0.48	4.57	7.48	0.09
Silty clay loam	0.34	0.21	0.46	1.77	8.02	0.25
Sandy clay	0.31	0.23	0.41	1.19	13.00	0.50
Silty clay	0.37	0.25	0.49	2.95	9.76	0.08
Clay	0.36	0.27	0.47	3.18	12.28	0.25

Field capacity, wilting point, porosity, saturated hydraulic conductivity (*K*_*sat*_), and *b* are taken from a lookup table^26^, which is provided with the VIC version 4 software documentation.

We set the variable infiltration capacity parameter ${b}_{infilt}=0.2$, the maximum baseflow fraction threshold ${d}_{s}=0.001$, and maximum soil moisture threshold ${w}_{s}=0.9$, their suggested values from the VIC documentation. These parameters, along with maximum baseflow velocity (*dsmax*) and soil depth, are typically calibrated. We set the baseflow curve exponent *c* = 2, the soil thermal damping depth *dp* = 4 m, soil density = 2685 kg/m^3^, surface roughness = 0.001 m, and snow roughness = 0.0005 m, also based on guidance from the VIC documentation. The soil moisture diffusion parameter *phi*_*s*_ is not used in the current version of VIC, so we set it to the no-data value (−999). The final few soil parameters — *dsmax*, initial soil moisture (*initm*), and bubbling pressure (*bubble*)— were calculated using the following equations, based on guidance from the VIC documentation.

1 $$dsmax=slope\ast {\bar{K}}_{sat}$$

2 $$initm=wc{r}_{fract}\ast porosity\ast {t}_{l}$$

3 $$bubble=0.32\ast expt+4.3$$

Equation (1) estimates *dsmax* for each grid cell as the product of soil-column average *K*_*sat*_ and land surface slope, which was calculated from the elevation data using the MATLAB® function *gradientm*^29^*g*. Equation (2), where *t*_*l*_ is the thickness of soil layer *l*, assumes that initial soil moisture is equal to the fractional soil moisture content at the critical point. Equation (3) calculates bubbling pressure as a function of *expt*, based on linear regression of bubbling pressure vs. *expt*^30^. Figures S2–S9 in the Supplementary Information show maps of each soil parameter. We assumed residual soil moisture, the amount of soil moisture that cannot be removed from the soil by drainage or evapotranspiration, was zero.

### Elevation bands

VIC uses an elevation band file (also called a snow band file) to account for subgrid heterogeneity in grid cell elevations. The assumption of uniform elevation over an entire grid cell can lead to modeling errors in mountainous regions, where higher topography is associated with cooler temperatures and higher precipitation rates. The elevation band file accounts for subgrid variability in topography by dividing each grid cell into a number of elevation bands, each of which is simulated separately. VIC adjusts temperature, pressure, and precipitation depending on the elevation in each band. We prepared an elevation band file with five elevation bands by comparing the 1/16° DEM used for the soil parameter file with a 30 arc-second DEM. Both DEMs were derived by aggregating MERIT data. For simplicity, we assumed precipitation was evenly distributed among elevation bands within a grid cell. The elevation band file is provided with the caveat that using elevation bands requires more computing power; users may wish to turn elevation bands on or off (via the VIC global parameter file) depending on their needs.

### Vegetation parameters

VIC-5 Classic uses a vegetation parameter file to define the fractional cover of different vegetation types within each grid cell and some of their physical properties. Other vegetation parameters are stored in the “vegetation library” file. (VIC-5 Image simply stores all parameters in a single “parameter” file.) The VIC-5 Classic vegetation parameter file consists of information about fractional cover of each land cover type in each grid cell, and their corresponding root zone depths and root fractions within each root zone. The vegetation parameter file can optionally include time-varying LAI, fractional canopy cover, and albedo data, but it is simpler to specify these in the vegetation library (at the cost of not representing some spatial heterogeneity).

**Fig. 3:**
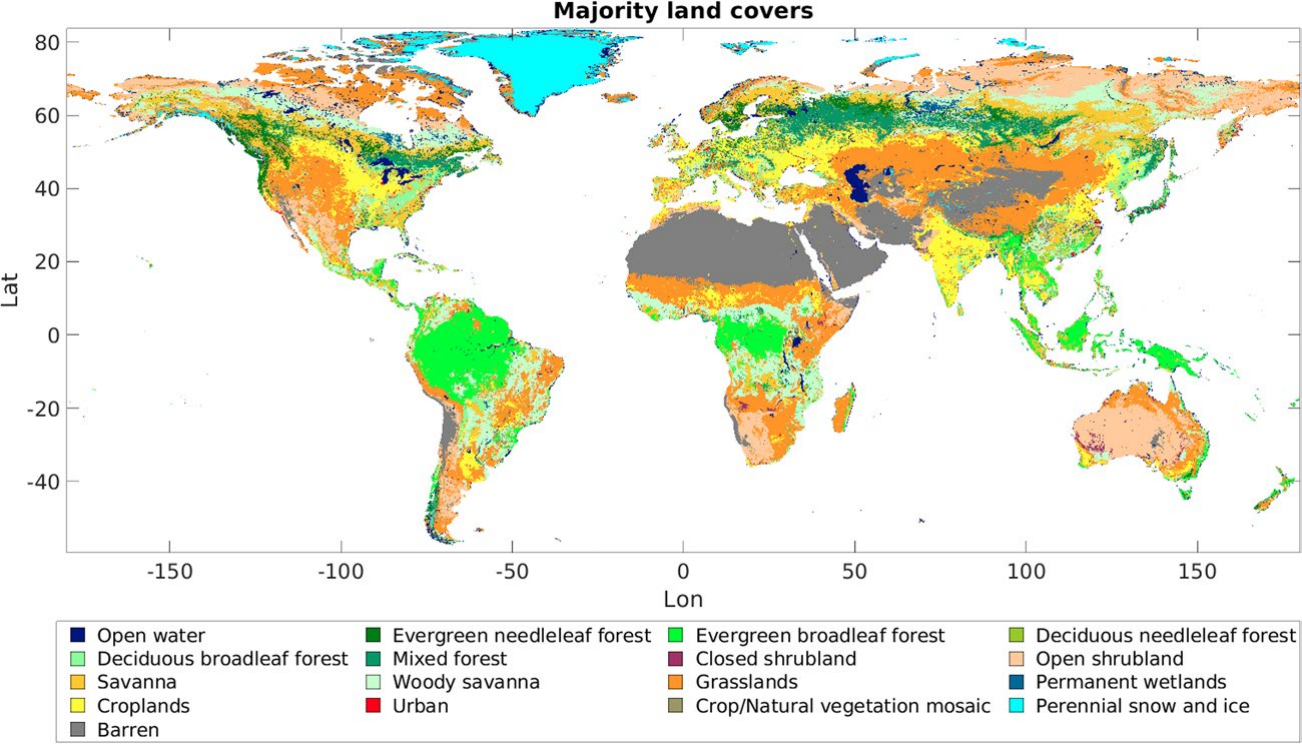
MODIS MCD12C1 majority land cover types (IGBP classifications). .

We used MODIS land cover data from the 0.05° MODIS MCD12C1 Collection 6 data product^31^ to assign fractional land cover values to each grid cell by calculating the average land cover for MCD12C1 observations over the 2017 calendar year. We chose 2017 because it was the most recent year with data in all the MODIS-based datasets used for this study, and there is very low interannual variability of land cover^32^ in MCD12C1 Collection 6. Figure 3 shows majority land cover types from the 2017 MCD12C1 observations.

Like all global land cover data products, MCD12C1 makes classification errors. Sulla-Menashe *et al.*^32^ reported 67% overall IGBP classification accuracy for 2001 land cover. Classification errors are more common in the “mixed” land covers, such as cropland/natural vegetation mosaic, shrublands, grasslands, and savannas. Fortunately for our purposes, the vegetation parameters for commonly-confused land covers tend to be fairly similar themselves, which reduces the impact of misclassification on land surface modelling results. For example, the LAI of open shrubland is not too different from the LAI of closed shrubland.

We calculated root fraction as a function of land cover class following the method of Zeng^33^, who defined the following formula (Eq. 4) for use in parameterizing land surface models:

4 $$Y=1-\frac{1}{2}\left({e}^{-ad}+{e}^{-bd}\right)$$

where *Y* = cumulative root fraction, *d = *depth, and *a* and *b* are empirical parameters defined by Zeng^33^ for each International Geosphere–Biosphere Programme (IGBP) land cover type, based on a rooting depth database compiled from more than 200 field surveys. We used this formula with depths of 0.1 m, 0.7 m, and *dr*, corresponding to three root zones. The value of *dr*, the maximum rooting depth for each IGBP land cover type, was taken from Zeng^33^. This method assumes that the depth and distribution of roots depends only on the land cover type; we assume that land cover type is the primary control on root characteristics. Table 3 shows root fractions and root zone depths for each IGBP land cover type.

**Table 3:** Root zone depths (m) and fraction of roots in each zone for IGBP land cover classes.

	Depth(1)	Depth(2)	Depth(3)	Fract(1)	Fract(2)	Fract(3)
Open water	0.1	0.6	0.8	0.44	0.45	0.11
Evergreen needleleaf forest	0.1	0.6	1.1	0.34	0.51	0.14
Evergreen broadleaf forest	0.1	0.6	2.3	0.32	0.44	0.23
Deciduous needleleaf forest	0.1	0.6	1.3	0.34	0.5	0.16
Deciduous broadleaf forest	0.1	0.6	1.3	0.31	0.52	0.17
Mixed forest	0.1	0.6	1.7	0.25	0.52	0.22
Closed shrublands	0.1	0.6	1.8	0.31	0.49	0.21
Open shrublands	0.1	0.6	2.4	0.33	0.43	0.24
Savanna	0.1	0.6	1.7	0.36	0.45	0.19
Woody savanna	0.1	0.6	1	0.37	0.5	0.13
Grasslands	0.1	0.6	0.8	0.44	0.45	0.11
Permanent wetlands	0.1	0.6	0.8	0.44	0.45	0.11
Cropland	0.1	0.6	0.8	0.33	0.55	0.12
Urban	0.1	0.6	0.8	0.44	0.45	0.11
Cropland/natural vegetation mosaic	0.1	0.6	0.8	0.33	0.55	0.12
Permanent snow and ice	0.1	0.6	0.8	0.44	0.45	0.11
Barren	0.1	0.6	3.3	0.22	0.46	0.31

Like previous large-scale VIC vegetation cover datasets, our vegetation parameter file neglects land cover change over time. However, it does have a few other advantages over past vegetation parameter datasets. The land cover classification used in the N2001 and L2013 VIC parameter sets is referred to as “UMD-NLDAS” because it is a modified version of the AVHRR-based University of Maryland (UMD) land cover product^34^. The UMD-NLDAS classification was modified for the North American Land Data Assimilation project (NLDAS^35^) to exclude open water, urban, and snow and ice land cover classes (see BV2019). VICGlobal uses 17 IGBP land cover classes, including urban, barren, perennial snow and ice, and inland water bodies, permitting better description of land cover variability than the 11 UMD-NLDAS classification.

### Vegetation library file

The vegetation library maps each land cover type to a set of vegetation parameters (Table 4). We adapted the LDAS vegetation library^36^ for use with the 17 IGBP land cover classes, taking monthly average LAI, fractional canopy cover (*fcanopy*), and albedo values obtained from recent MODIS data products. We set architectural resistance (*r*_0_) and minimum stomatal resistance (*r*_*min*_) to values from literature (described below). The rest of the parameters, which are described in the N2001 paper, were left to their original LDAS vegetation library values. This section describes how we estimated LAI, *fcanopy*, albedo, *r*_0_, and *r*_*min*_, and how we transferred the remaining parameters from the 11 UMD-NLDAS land cover classes to the 17 IGBP land cover classes.

**Table 4:** VIC model parameters for the vegetation library file.

Parameter	Description	Source
Overstory	Flag for whether the land cover type has an overstory	LDAS^36^
*R*_*0*_ (s/m)	Architectural resistance	SECHIBA^42^
*R*_*min*_ (s/m)	Minimum stomatal resistance	SiB^44^
LAI	Monthly average leaf-area index	2017 GLASS LAI^37–39^
Canopy cover fraction	Monthly average partial vegetation cover fraction	2017 MODIS NDVI^40^
Albedo	Monthly average albedo	2017 GLASS albedo^37–39^
Roughness length (m)	Average roughness length	LDAS^36^
Displacement height (m)	Average displacement height	LDAS^36^
WindH	Wind measurement height	LDAS^36^
RGL	Minimum incoming shortwave radiation for transpiration to occur	LDAS^36^
Solar attenuation	Radiation attenuation factor	LDAS^36^, set to 0.5
Wind attenuation	Wind attenuation factor through the overstory	LDAS^36^, set to 0.5
Trunk fraction	Ratio of total tree height that is trunk	LDAS^36^, set to 0.2

Parameters remapped from UMD-NLDAS to IGBP classification, following BV2019, are assigned the source “LDAS.”

We used MODIS observations from the year 2017 to calculate monthly average LAI, *fcanopy*, and albedo for each IGBP land cover type. We calculated LAI and albedo from the MODIS-based Global LAnd Surface Satellite dataset (GLASS^37–39^) and *fcanopy* from NDVI observations (MCD13C1^40^) The expression used for *fcanopy* follows BV2019:

5 $$fcanopy={\left(\frac{NDVI-NDV{I}_{min}}{NDV{I}_{max}-NDV{I}_{min}}\right)}^{2}$$

where *NDVI*_*min*_ and *NDVI*_*max*_ are the minimum and maximum values of NDVI observed for that month. Monthly LAI, *fcanopy*, and albedo values were calculated by averaging over all grid cells of the same land cover type, counting only cells that were at least 90% homogenous, to avoid noise from grid cells with multiple land covers. Excepting perennial snow and ice land cover, the vegetation parameters in the VIC vegetation library should describe snow-free vegetation. Therefore, before calculating LAI, *fcanopy*, and albedo for each land cover class, we used fractional snow cover data from MOD10CM^41^, a global 0.05 degree monthly snow cover dataset, to exclude grid cells with more than 90% snow cover. Additionally, we set albedo to 0.05 for open water, and we set LAI and *fcanopy* to 0 for open water and perennial snow and ice.

The resistances *r*_*min*_ and *r*_0_ play a role in determining how much plant transpiration occurs. Higher resistance means less transpiration. Stomatal resistance is resistance to the release of water through the plant stomata, and architectural resistance is the aerodynamic resistance between the leaves and the canopy top^42^. Two sets of resistance parameters have been used in past large-scale VIC implementations. N2001 ran VIC over the entire globe using *r*_*min*_ values adapted from Dorman and Sellers’ global database of *r*_*min*_ values^43^ computed using the Simple Biosphere Model^44^ (SiB). The Nijssen *et al.*^45^* r*_0_ values were taken from Ducoudre *et al*.’s SECHIBA land surface parameterization^42^. The other set of *r*_*min*_ and *r*_0_ parameters are those used in the LDAS vegetation library and in studies such as Livneh *et al.*^12^. This set of *r*_*min*_ values comes from Mao *et al.*^46^ and Mao and Cherkauer^47^. We used the *r*_*min*_ values from SiB^44^ and the *r*_0_ values from SECHIBA^42^ for VICGlobal as they appeared to be the better documented values.

For the other parameters in the vegetation library file (displacement height, roughness length, etc.), we assigned values using the existing LDAS vegetation library. Since there are 17 IGBP land cover classes, and only 11 UMD-NLDAS land cover classes in the LDAS vegetation library, we re-assigned some IGBP land cover classes to take the parameters of UMD-NLDAS land cover classes. We remapped barren land, permanent wetlands, snow and ice, urban land, and water bodies to take the parameters of “grasslands” from the LDAS vegetation parameter file. While the characteristics of the barren, snow and ice, urban, and water land cover types clearly differ from those of grasslands, their low LAI and *fcanopy* values, corresponding to sparse vegetation, essentially “turns off” the other vegetation parameters in the VIC model, as pointed out by BV2019. The other remappings were more straightforward. Croplands and croplands/natural vegetation mosaics inherited values from “croplands,” savannas became “wooded grasslands,” and woody savannas became “woodlands.” We were thus able to assign vegetation parameter values to the each of the 17 IGBP land cover classes.

**Fig. 4:**
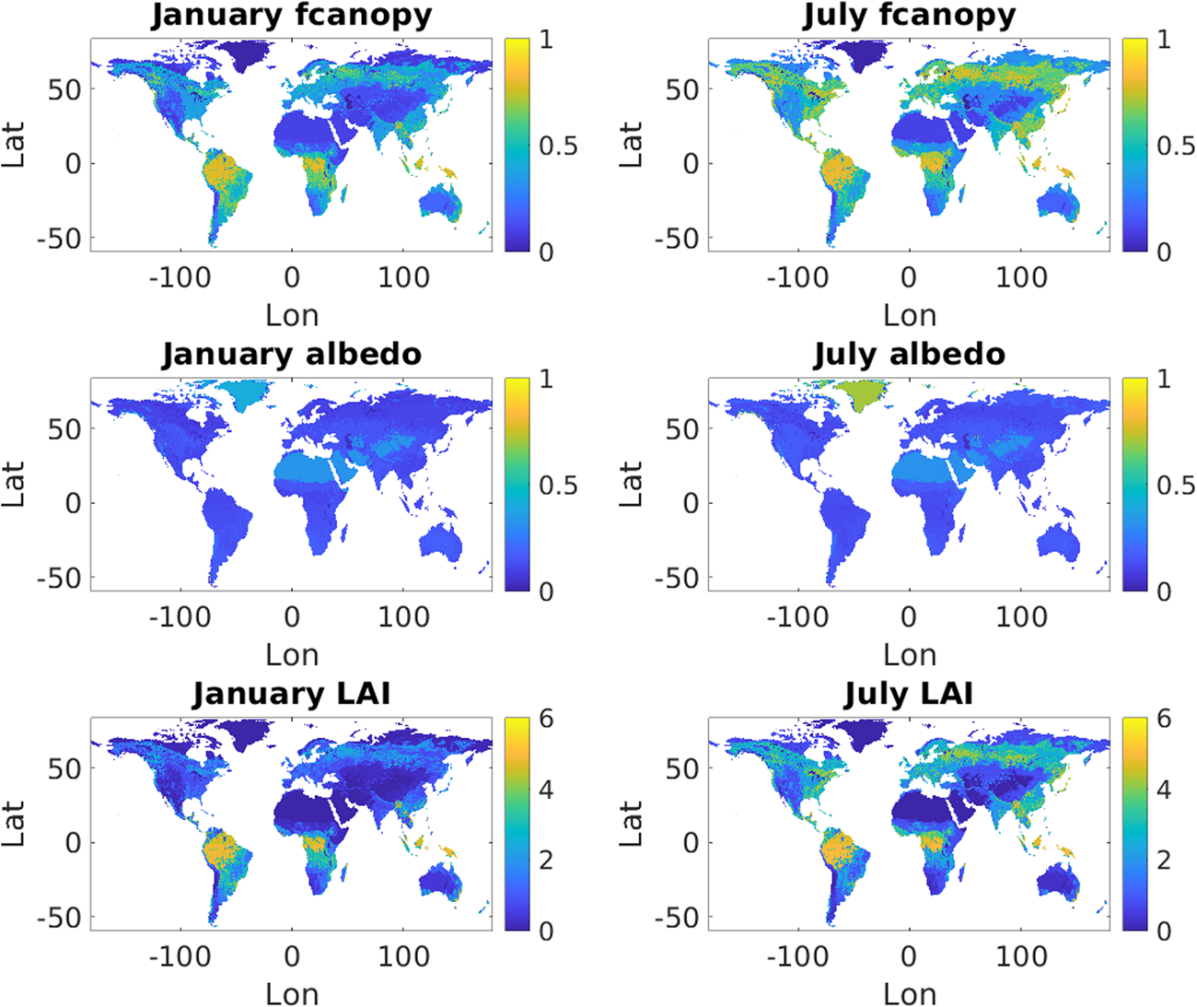
Maps of leaf-area index, albedo, and fractional canopy cover values. Parameter values have been averaged over the 17 IGBP land cover classes using area-based weighting.

To calculate global average time series of seasonally-varying vegetation parameters would be of limited interest as the seasonal cycle would average out across the equator. Therefore, we calculated average monthly *fcanopy*, LAI, and albedo for each vegetation type in each hemisphere, and we developed two separate vegetation library files: one for the northern hemisphere and one for the southern hemisphere. Maps of January and July LAI, *fcanopy*, and albedo are shown in Fig. 4. For illustrative purposes, the parameter values in this figure have been averaged over the 17 IGBP land cover classes using area-based weighting. Figures S14–S19 show maps of the remaining vegetation parameters. Figures S20–S22 show the cycle of LAI, fractional canopy cover, and albedo for each vegetation type, averaged separately over each hemisphere.

## Data Records

Soil and vegetation parameters for the VIC model are available for download at Zenodo^48^ in NetCDF format for version 5 of the VIC model. The files are stored as zip archives. *parameters_classic.zip* contains ASCII text files with soil parameters, vegetation parameters, elevation bands, and two vegetation library files — one of the northern hemisphere and one for the southern hemisphere — for VIC-5 Classic. *parameters_global.zip* contains a NetCDF “parameter” file with all the soil and vegetation parameters described above and a NetCDF “domain” file describing the VICGlobal domain (all land mass between 60°S and 85°N) for VIC-5 Image. MATLAB® codes for subsetting either set of parameters from the entire VICGlobal extent to a subregion of interest. Additionally, parameter and domain files pre-subsetted to North America, South America, Africa, Eurasia, and Oceania are available for download.

## Technical Validation

### Streamflow and snow-water equivalent in the Upper Colorado Basin

Having created input files for the VIC model, we tested the parameters in a large, well-studied river basin. We used the VICGlobal parameters to run VIC in water balance mode over the Upper Colorado River Basin (UCRB), a 293,600 km^2^ basin in the western United States. We ran VIC once using the VICGlobal parameters and once using the L2013 parameters. Both simulations used the meteorological forcing data from L2013, at a six-hourly timestep, in water balance mode, for the 6-year period from Oct. 1, 2005 to Sept. 30, 2011.

**Fig. 5:**
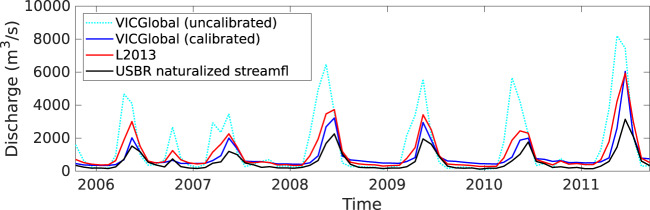
Monthly discharge estimates at Lee’s Ferry (the outlet of the Upper Colorado River Basin). The dotted cyan line shows the uncalibrated VICGlobal estimate, the dark blue line shows the calibrated VICGlobal estimate, the red line shows the L2013 estimate, and the black line shows the U.S. Bureau of Reclamation naturalized streamflow data.

We compared estimated streamflow from the VICGlobal and L2013 simulations with naturalized streamflow estimates from the U.S. Bureau of Reclamation^49^ (USBR) at Lees Ferry, Arizona (Fig. 5). Naturalized flow is measured streamflow adjusted for the effects of reservoir storage and management and consumptive uses such as irrigation. We compared our VIC model outputs with naturalized streamflow because our VIC implementation does not simulate consumptive water use or reservoir storage. Due to differences in soil and vegetation parameters between the two sets of input files, there are notable differences in the hydrographs from each simulation. Relative to the L2013 results, the uncalibrated VICGlobal peak flows’ timing is too early and their magnitude is too high. This is expected given that the L2013 parameters have been calibrated to get a good match to gauge data.

**Fig. 6:**
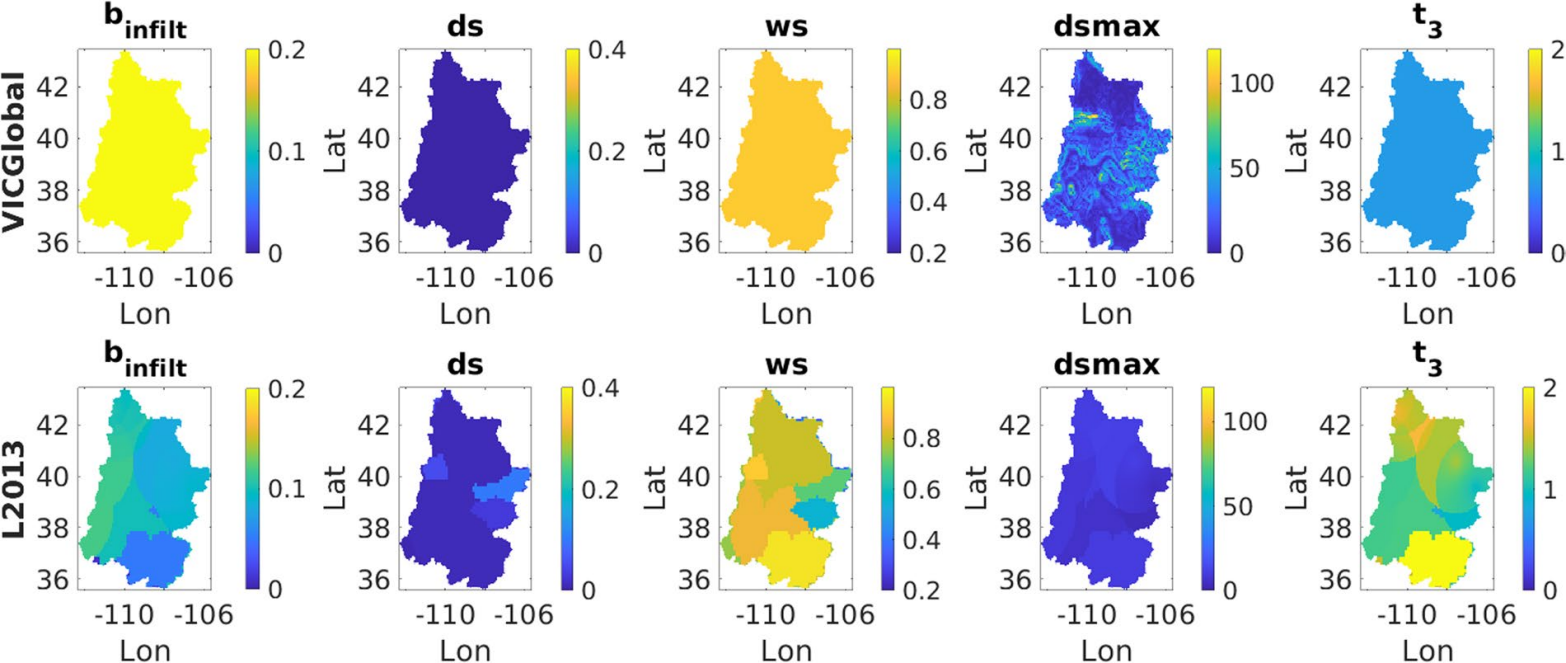
Maps of VICGlobal and L2013 soil parameters in the Upper Colorado River Basin.

To understand the cause of this mismatch, we examine seven commonly calibrated soil parameters, which are difficult or impossible to estimate from measurements: *ds*, *ws*, *dsmax*, *b*_*infilt*_, and the thicknesses of each soil layer (*t*_1_, *t*_2_, *t*_3_). Taking a closer look at these soil parameters in the UCRB (Fig. 6), we see that the infiltration capacity parameter *b*_*infilt*_ is considerably higher in the VICGlobal parameters than it is in the L2013 parameters, which would tend to cause higher runoff rates. *ds* is lower for VICGlobal than for L2013, so nonlinear baseflow occurs at a lower fraction of *dsmax*, tending to make baseflow peaks occur earlier. *dsmax* is considerably higher for VICGlobal than for L2013 in much of the UCRB, so the maximum baseflow rate is higher for VICGlobal. Finally, the thicker soil layers in L2013 mean that more water can infiltrate into the soil before baseflow occurs. Table 5 describes each of the seven calibration parameters and their influence on VIC model outputs. VIC users seeking more guidance on calibrating soil parameters should consult the VIC model documentation and relevant literature^50–52^. We calibrated the VICGlobal parameters *b*_*infilt*_, *dsmax*, and *t*_3_, the same parameters calibrated by L2013, to get a good match between predicted and observed (USBR naturalized) streamflow. However, decreasing *b*_*infilt*_ on its own was not enough to reduce the high runoff estimates produced by the VICGlobal parameters (VIC’s sensitivity to *b*_*infilt*_ depends on the water-holding capacity of the upper two soil layers). By introducing the thickness of the second soil layer *t*_2_ as a fourth calibration parameter, we were able to reduce runoff and increase transpiration. The final set of calibrated parameters was ${b}_{infilt}=0.038$, *dsmax* = 0.60 mm⁄day, *t*_2_ = 1.5m, and *t*_3_ = 1.6m.

**Table 5:** Commonly calibrated soil parameters in the VIC model and their effects on model outputs.

	Description	Effect	Min	Max
*ds*	Fraction of dsmax where non-linear baseflow begins.	For a given soil moisture content, the linear baseflow term increases with *ds*, while the nonlinear term decreases.	0	1
*dsmax*	Maximum baseflow velocity in lowest soil layer	Baseflow increases proportionally to *dsmax*.	0	About 30 mm/day
*ws*	Fraction of max soil moisture at which nonlinear baseflow occurs.	Higher value raises the water content required for rapid increase in baseflow (nonlinear baseflow term), resulting in delayed baseflow peaks.	0	1
*b*_*infilt*_	Infiltration capacity parameter. Defines the shape of the VIC curve.	Higher value of *b* gives lower infiltration and thus higher surface runoff.	0	0.4
*t*_*1*_	Thickness of upper soil layer	Thicker soil means more water can be stored before baseflow occurs. Also, more evaporation occurs if there is more water stored in the soil.	0.01 m	0.5 m
*t*_2_	Thickness of middle soil layer	Controls water availability for transpiration. Thicker soils store more water.	0.05 m	1 m
*t*_3_	Thickness of bottom soil layer	Thicker soil means more water can be stored before baseflow occurs. Controls water availability for baseflow.	0.5 m	2.5 m

The baseflow equations are found in Liang *et al*. (1994).

For this analysis, we used manual calibration because the model run time made automated methods, which require hundreds to thousands of model runs, impractical. We used a custom MATLAB® application — a graphical user interface for running the VIC model, tuning its parameters, and displaying its outputs — to assist with manual calibration. We assumed the calibrated parameters were uniform over the basin. In addition to calibration by trial and error, VICGlobal users may also wish to explore automated calibration methods such as the Shuffled Complex Evolutionary algorithm^53^ (SCE-UA) or Dynamically-Dimensioned Search^54^ (DDS) when practical.

**Fig. 7:**
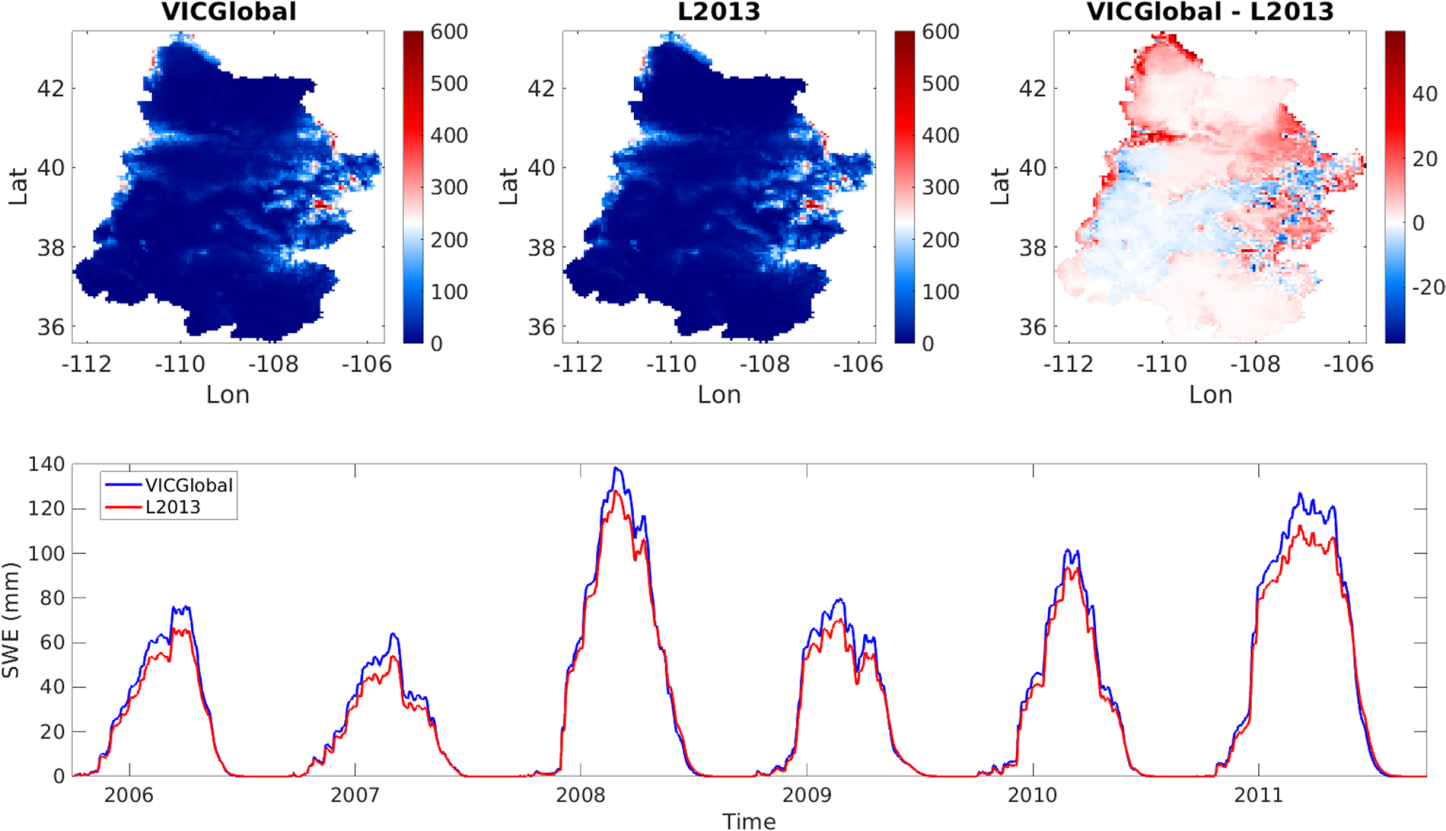
Time-average snow-water equivalent (SWE) maps (**a**–**c**) and basin-average SWE time series (**d**) for the Upper Colorado River Basin comparing simulations using the L2013 and VICGlobal parameters.

The calibrated VICGlobal simulation outperformed the L2013 simulation, with a Kling-Gupta efficiency^55^ (KGE) of 0.24, compared to −0.26 for L2013 and −1.7 for the uncalibrated VICGlobal simulation. We also compared simulated snow-water equivalent (SWE) between the VICGlobal and L2013 simulations. Spatial patterns of simulated SWE were consistent between the two simulations (Fig. 7a–c), as were patterns of snow accumulation and melt (Fig. 7d). VICGlobal SWE was 3 mm higher than L2013 SWE, on average. Snow sublimation, including canopy sublimation, was higher for L2013 than for VICGlobal, which helps explain the slight overestimate of SWE by VICGlobal relative to L2013. Overall, VICGlobal is able reproduce the timing, magnitude, and spatial pattern of L2013-simulated SWE in the UCRB, with no need for parameter calibration.

### Water balance in 12 unregulated CONUS basins

**Fig. 8:**
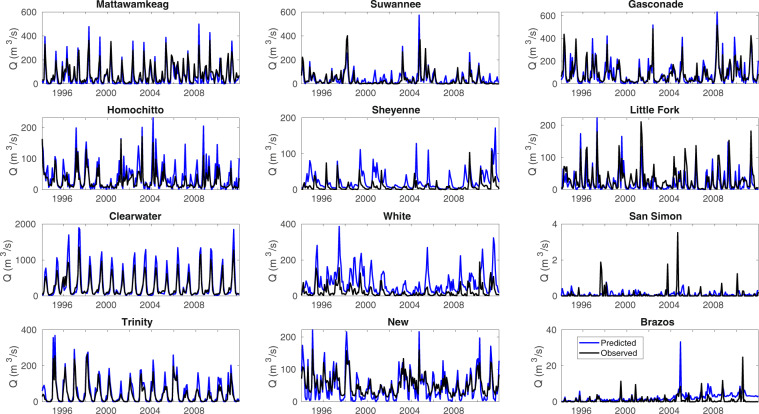
Monthly discharge predictions for 12 CONUS basins compared to USGS reference gauge measurements.

Beyond the Upper Colorado Basin, we evaluated the VICGlobal parameters’ potential for modelling the water and energy balance in 12 basins, ranging from 1500–25000 km^2^, chosen for good spatial coverage of the CONUS. Modelled discharge was compared with monthly observations at USGS reference stream gages^56^ at each basin outlet; we used the DDS method to calibrate *b*_*infilt*_, *dsmax*, *t*_2_, and *t*_3_. (These basins are small enough for automatic calibration to be practical.) The calibration was performed with L2013 meteorological forcing data, for calendar year 1993, with the VIC model run from 1990 – 1992 as spin-up. After 500 model evaluations (50 for the Clearwater River), the average discharge calibration KGE was 0.47, with a maximum of 0.83 for the Clearwater River in Idaho and a minimum of 0.01 for the White River in Arkansas. Using the calibrated parameters, we performed a validation run from 1994–2011. Table 6 shows goodness of fit between modelled and measured discharge for the 18-year validation run. Figure 8 shows discharge plots for each basin over the validation period. Good matches can be seen for the Clearwater, New, Homochitto, Mattawamkeag, Gasconade, Trinity, and Little Fork rivers, while the White, Sheyenne, Brazos, and San Simon rivers did not respond well to calibration, suggesting that parameters other than the four calibrated here are to blame. See e.g. Demaria *et al.*^50^ for more insight on VIC calibration.

**Table 6:** Goodness of fit metrics for the 1994–2011 validation run over 12 CONUS basins.

	Calibration (1993)		Validation (1994–2011)		DA (km^2^)
	KGE	RMSE	KGE	RMSE	
Clearwater River	0.83	167	0.50	214	14268
Homochitto River	0.72	60	0.49	58	2073
Suwannee River	0.71	42	0.37	81	6136
New River	0.69	38	0.57	47	2952
Mattawamkeag River	0.64	86	0.70	66	3676
Gasconade River	0.62	235	0.72	115	8265
Sheyenne River	0.60	9	−0.87	28	7582
Trinity River	0.46	76	0.51	56	1980
Little Fork River	0.24	43	0.52	44	4383
San Simon Wash	0.12	1	−0.04	1	1482
Brazos River	0.05	1	−0.65	10	3348
White River	0.01	60	−1.47	117	25791

### Surface radiation budget validation with SURFRAD

To evaluate how well the (uncalibrated) VICGlobal parameters simulate the surface radiation balance, we ran VIC over six SURFRAD^57^ sites, using soil parameters from the 1/16° grid cell containing the SURFRAD sites. We ran hourly simulations in energy balance mode from 1995–2011, with 1994 as a spin-up year. Meteorological inputs taken from meteorological stations at the sites provided input data for the model, except for precipitation, which we took from L2013.

**Fig. 9:**
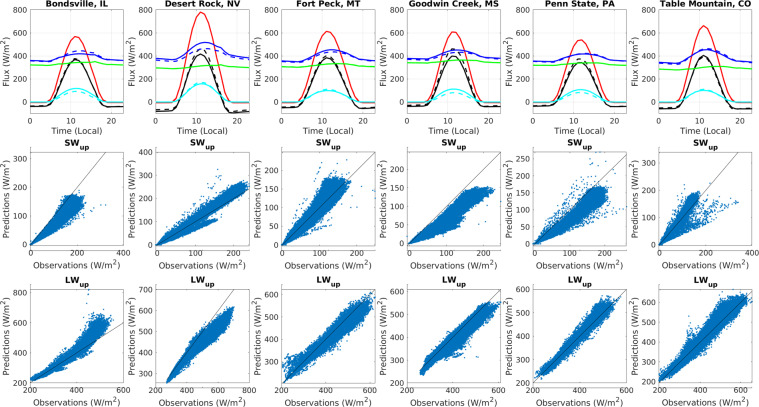
First row: Hourly modelled and observed net radiation, upwelling longwave, downwelling longwave, upwelling shortwave, and downwelling shortwave radiation from 1995–2011 for six SURFRAD sites in the CONUS. Dashed lines show predictions, while solid lines show observations. Second and third rows: scatterplots of predicted vs. observed upwelling shortwave and longwave radiation, with one-to-one lines shown in black. Key: Black = net radiation, red = downwelling shortwave, cyan = upwelling shortwave, green = downwelling longwave, blue = upwelling longwave.

The first row of Fig. 9 shows modelled and observed net radiation, upwelling longwave, downwelling longwave, upwelling shortwave, and downwelling shortwave radiation averaged over each day from 1995–2011 for six SURFRAD sites in the CONUS. Downwelling shortwave and longwave radiation predictions similar to the observations because the SURFRAD data were used as inputs for the VIC model (but not identical because precipitation inputs were taken from L2013 due to lack of ground measurements at the sites). There is a positive bias for net radiation resulting from a slight low bias for upwelling shortwave radiation. Overall, the bias is small.

The second and third rows of Fig. 9 show scatterplots of predicted vs. observed upwelling shortwave and longwave radiation, with one-to-one lines shown in black. The correlation between predicted and observed upwelling shortwave and longwave radiation is close to 1 (ranges from 0.96–0.99 for all sites). Running VIC with VICGlobal parameters allows simulation of upwelling longwave radiation with an RMSE of 25 W/m^2^ and RMSE of 15 W/m^2^ for upwelling shortwave radiation. In Fig. 9, we have excluded data at times when snow covers the ground to address the scale-issue — the spatial scale of the VIC simulations (a 1/16° grid cell) is much larger than that of the SURFRAD measurement — because of snow’s large role in determining upwelling solar radiation, we excluded times when either the VIC model or SURFRAD measurements had snow on the ground using an albedo threshold of 0.4; none of the VICGlobal albedos for non-snowy land surfaces are this large.

## Usage Notes

We have described VICGlobal, a globally-consistent 1/16° VIC parameter dataset with soil and vegetation parameters derived from the latest satellite-based remote sensing datasets (MODIS and MERIT, which is based on SRTM data) and *in-situ* soil data from the FAO HWSD. In addition to its higher resolution, VICGlobal has an advantage over previous global VIC setups due to its inclusion of seasonally-varying fractional canopy cover, LAI, and albedo, and because it explicitly accounts for barren, wetland, open water, and perennial snow and ice land covers. VICGlobal is provided in geographic coordinates, referenced to the WGS84 ellipsoid and datum.

VICGlobal has a few limitations. Its parameters are uncalibrated, so users must calibrate sensitive yet hard-to-measure parameters such as soil depth and the variable infiltration capacity parameter to get a good match between simulated and observed discharge. Several of the vegetation parameters, such as roughness length and displacement height, are assumed constant in time, even though realistically these parameters change as vegetation blooms and senesces throughout the year. And while we believe our monthly, hemisphere-average fractional canopy cover, LAI, and albedo are a major improvement over past global datasets, the most realistic parameter set would have them vary from grid cell to grid cell, even for the same vegetation type. Despite its limitations, we hope that VICGlobal, with its relatively high spatial resolution, wide coverage, and easy availability will be a valuable resource for VIC users.

## Supplementary information

Supplementary Information

## Data Availability

The scripts used to create the VICGlobal data set can be found on the corresponding author’s Github page (https://github.com/jschap1/vicglobal-prep and https://github.com/jschap1/vegpar). The VICGlobal parameters were subset to the Upper Colorado River Basin using the subsetting codes included with the VICGlobal dataset, archived on Zenodo^48^.
