# Supplementary material for: A near-global, high resolution land surface parameter dataset for the variable infiltration capacity model
Source: Sci Data. 2021 Aug 11;8:216. doi: 10.1038/s41597-021-00999-4 (PMC8357956; doi:10.1038/s41597-021-00999-4)
Supplement: Supplementary file 1 — Supplementary Information [file 41597_2021_999_MOESM1_ESM.docx]

**Supplementary information for “A near-global, high resolution land surface parameter dataset for the Variable Infiltration Capacity model”**

### Authors

Jacob R. Schaperow^1^, Dongyue Li^2^, Steven A. Margulis^1^, Dennis P. Lettenmaier^2^

**Affiliations**

1. Department of Civil and Environmental Engineering, University of California, Los Angeles 90095 U.S.A.

2. Department of Geography, University of California, Los Angeles 90095 U.S.A.

corresponding author: Jacob R. Schaperow (jschap@g.ucla.edu)

This Supplementary Information document includes:

- A plot of the resampled 1/16° MERIT DEM (Figure S1) and plots of each spatially-varying soil parameter (Figures S2-S13).
- Plots (Figures S14-S19) of vegetation parameters not shown in the main text. The vegetation parameters have been averaged over the 17 IGBP land cover classes.
- Plots (Figures S20-S22) of the monthly LAI, albedo, and fractional canopy cover for each of the 17 IGBP land cover types calculated based on several MODIS data products, described in the main text.


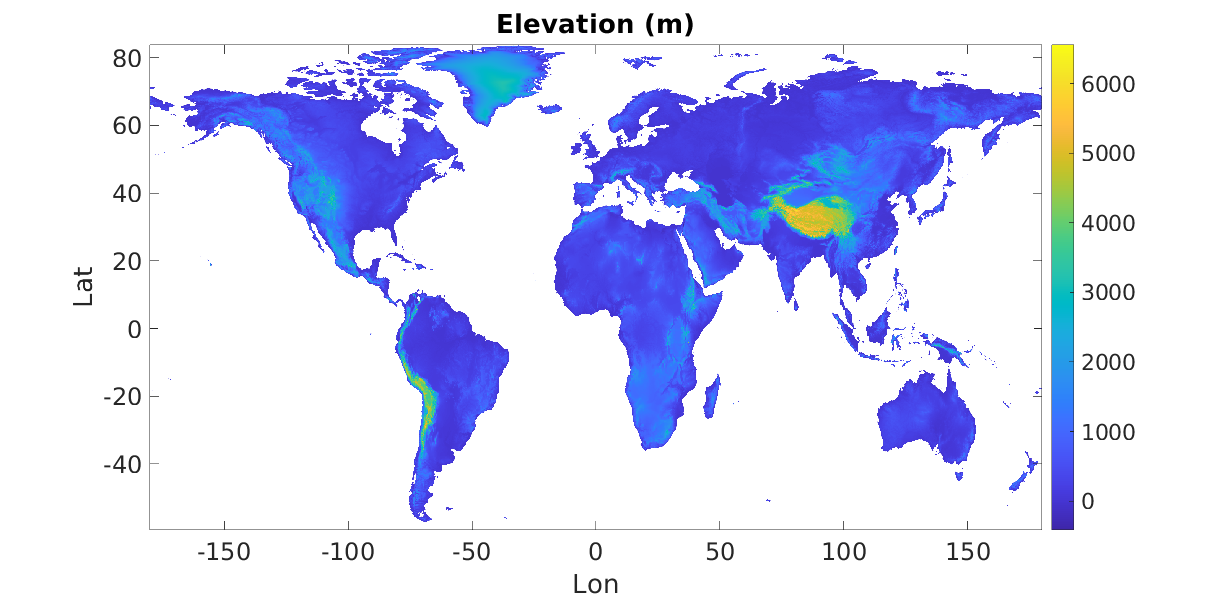


Figure S1. Elevation map showing the MERIT DEM resampled to 1/16° resolution. The spatial domain covers all the world’s land, except Antarctica.


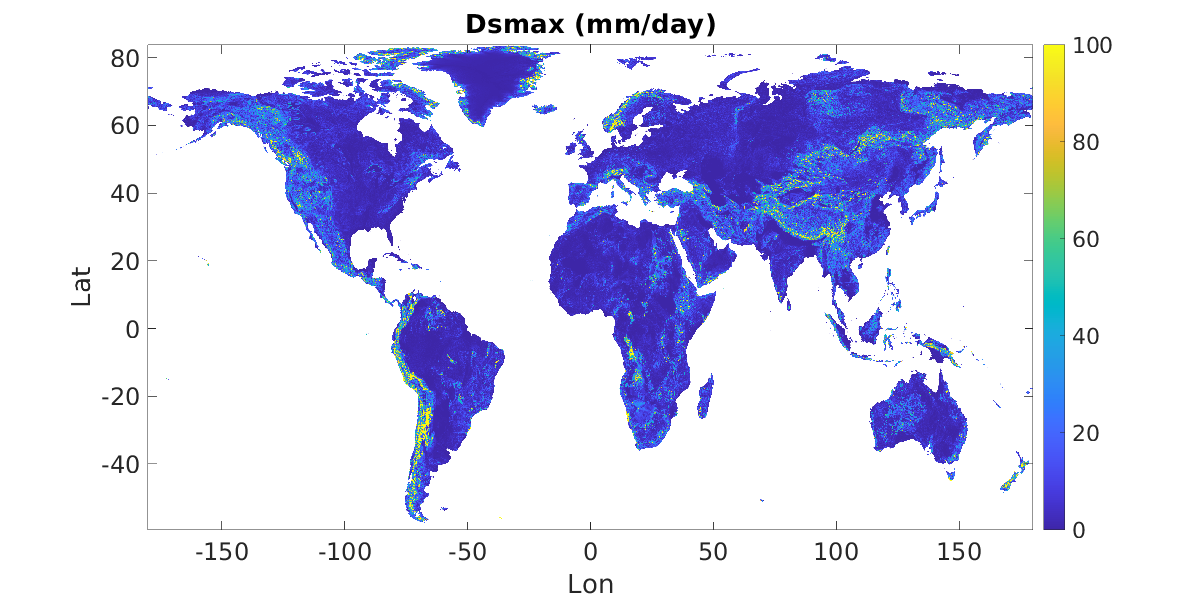


Figure S2. Maximum baseflow velocity *dsmax*. Values greater than 100 mm/day have been censored to show spatial variability.


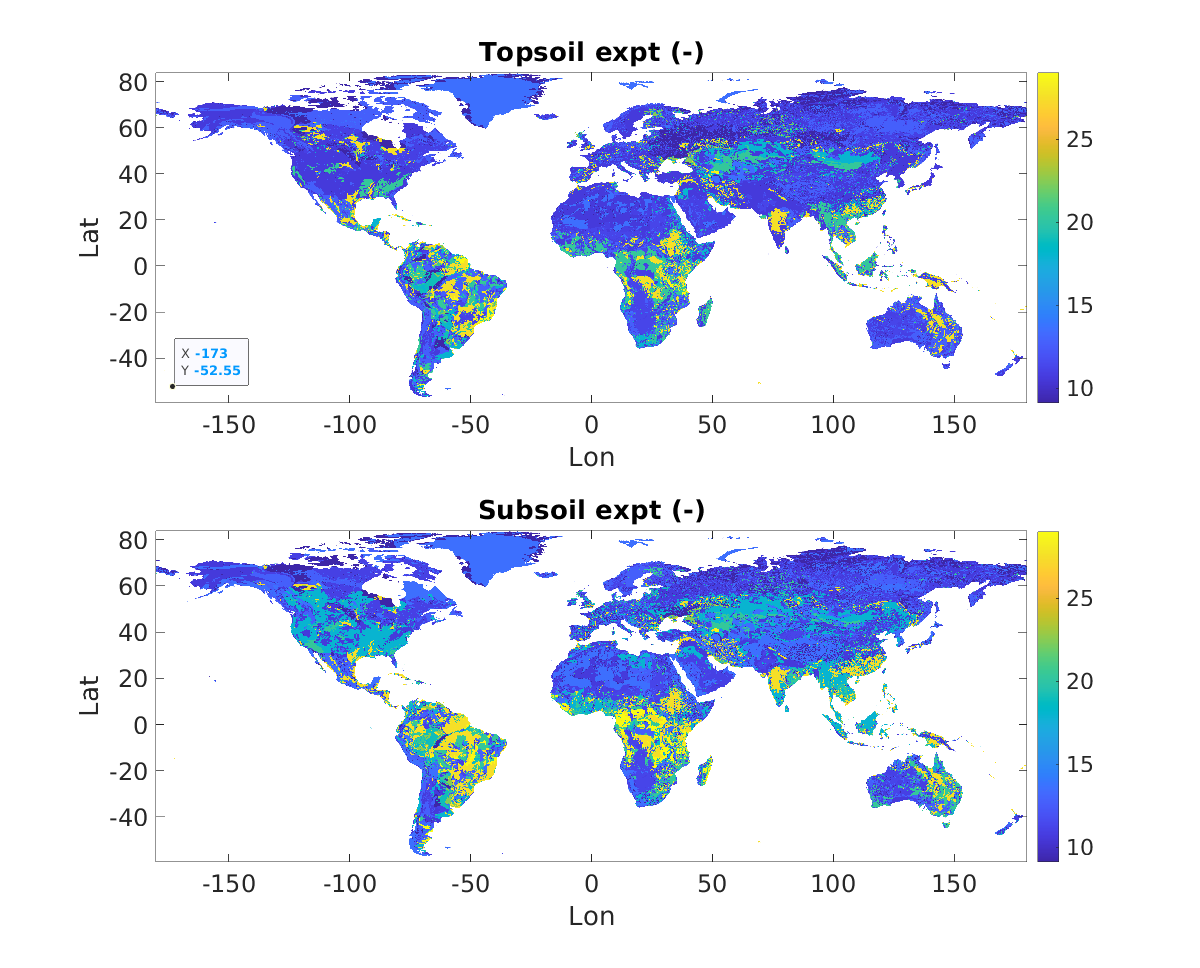


Figure S3. The exponent (*expt*) in Campbells’s equation for hydraulic conductivity, calculated based on the FAO’s Harmonized World Soils Database (HWSD). Topsoil values represent the first 30 cm of the soil column, and subsoil values represent soil from 30-100 cm deep.


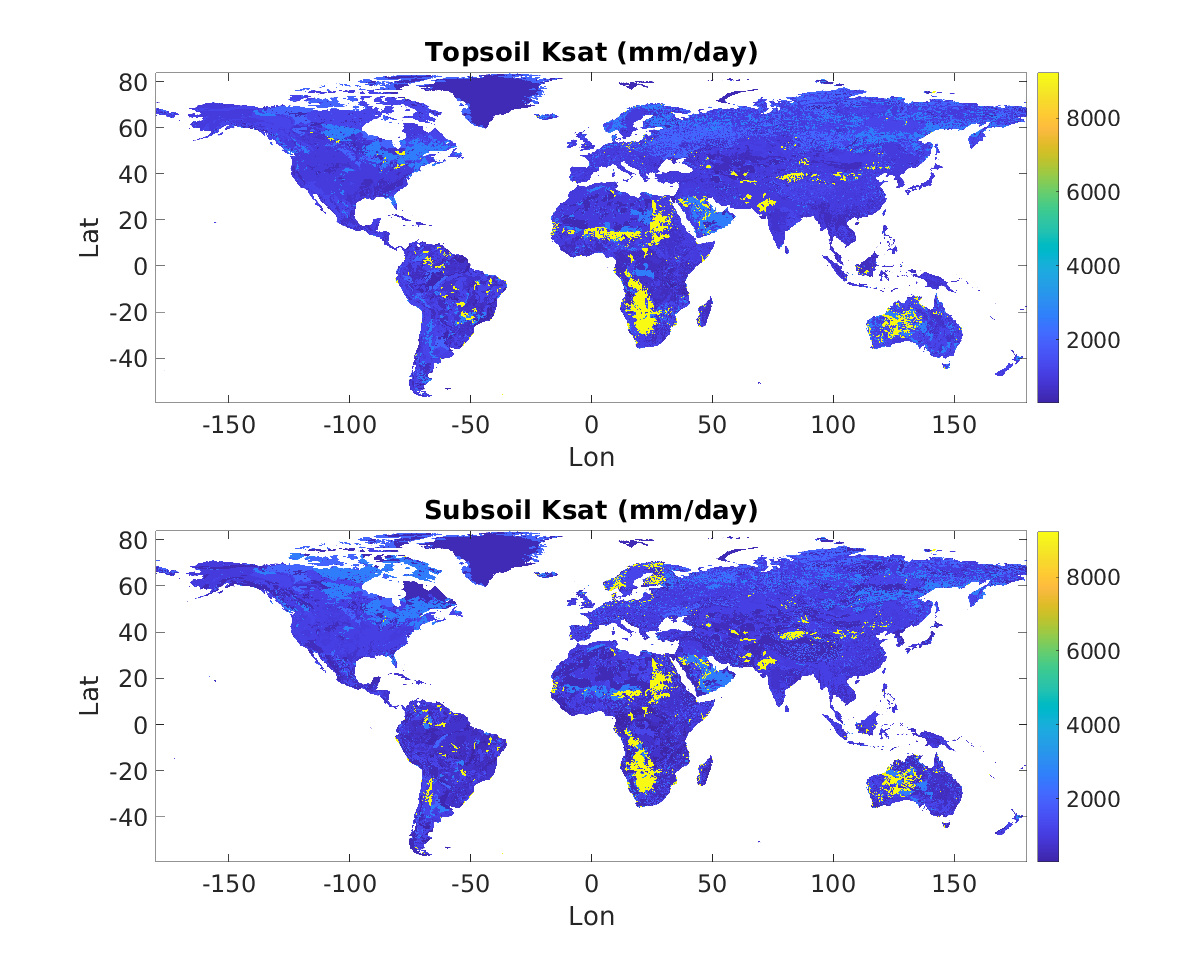


Figure S4. Saturated hydraulic conductivity (*K_sat_*) for topsoil and subsoil, calculated based on HWSD data.


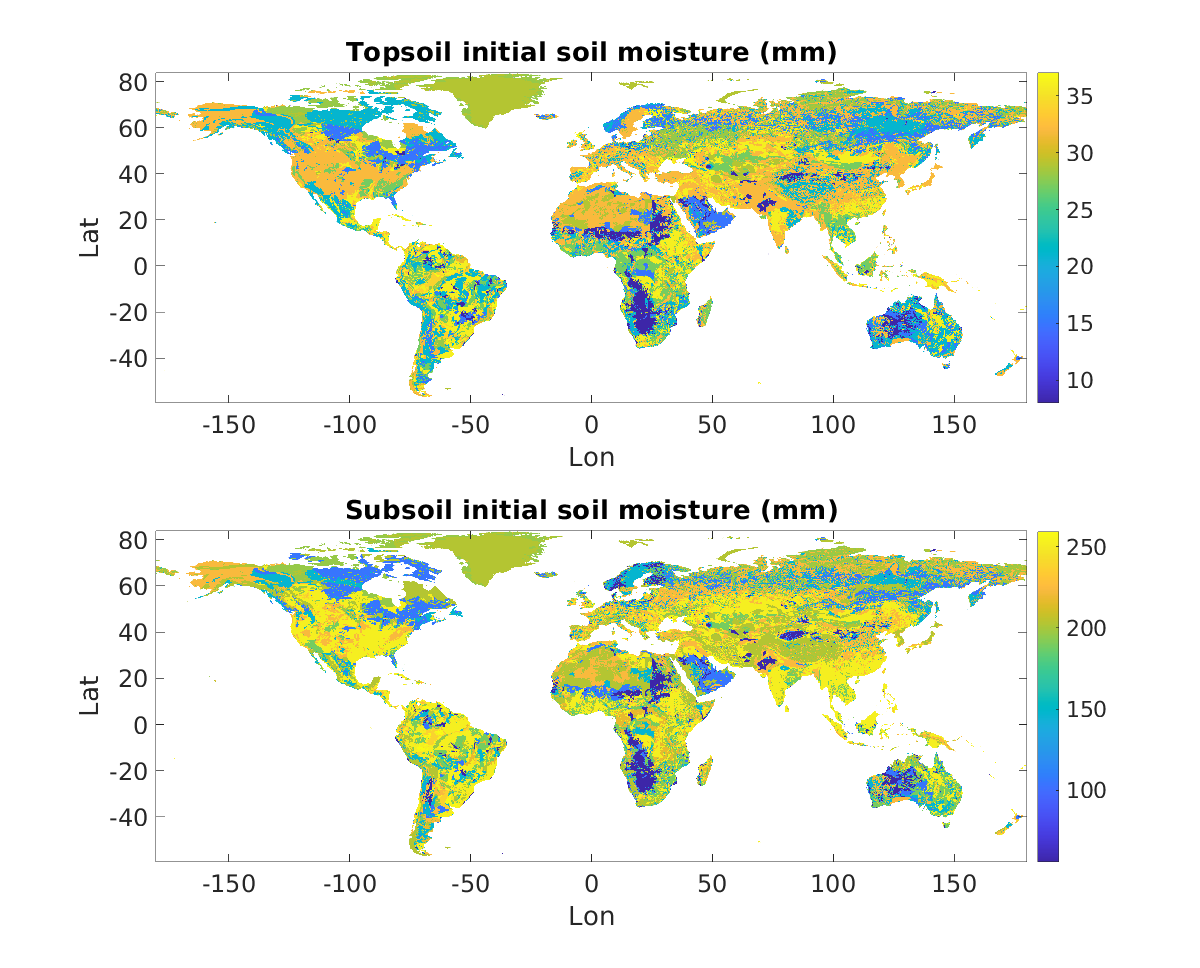


Figure S5. Initial soil moisture for topsoil and subsoil, calculated based on HWSD data, assuming the soil is initially at the critical point.


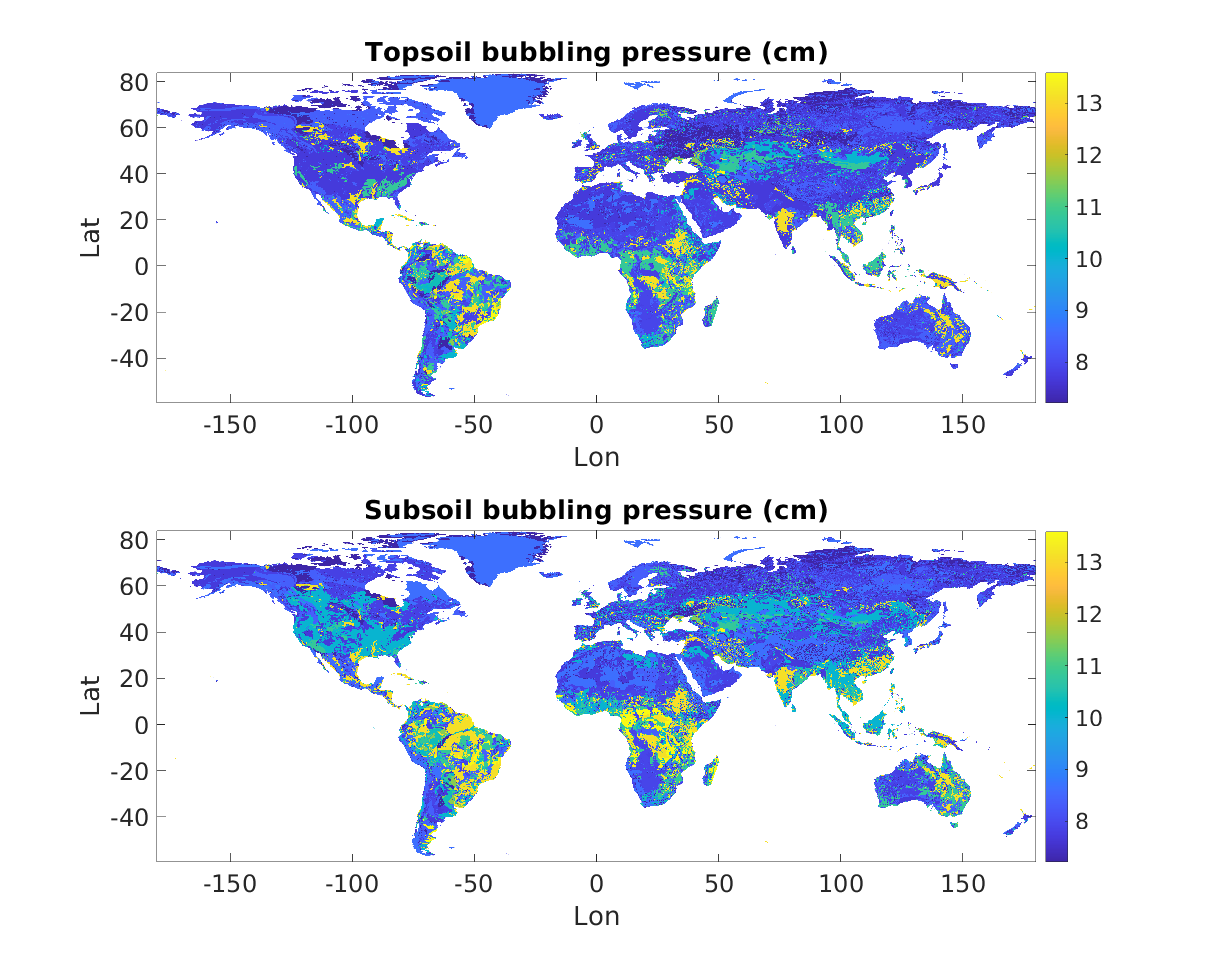


Figure S6. Bubbling pressure, calculated as a function of the Campbell’s equation exponent, expt.


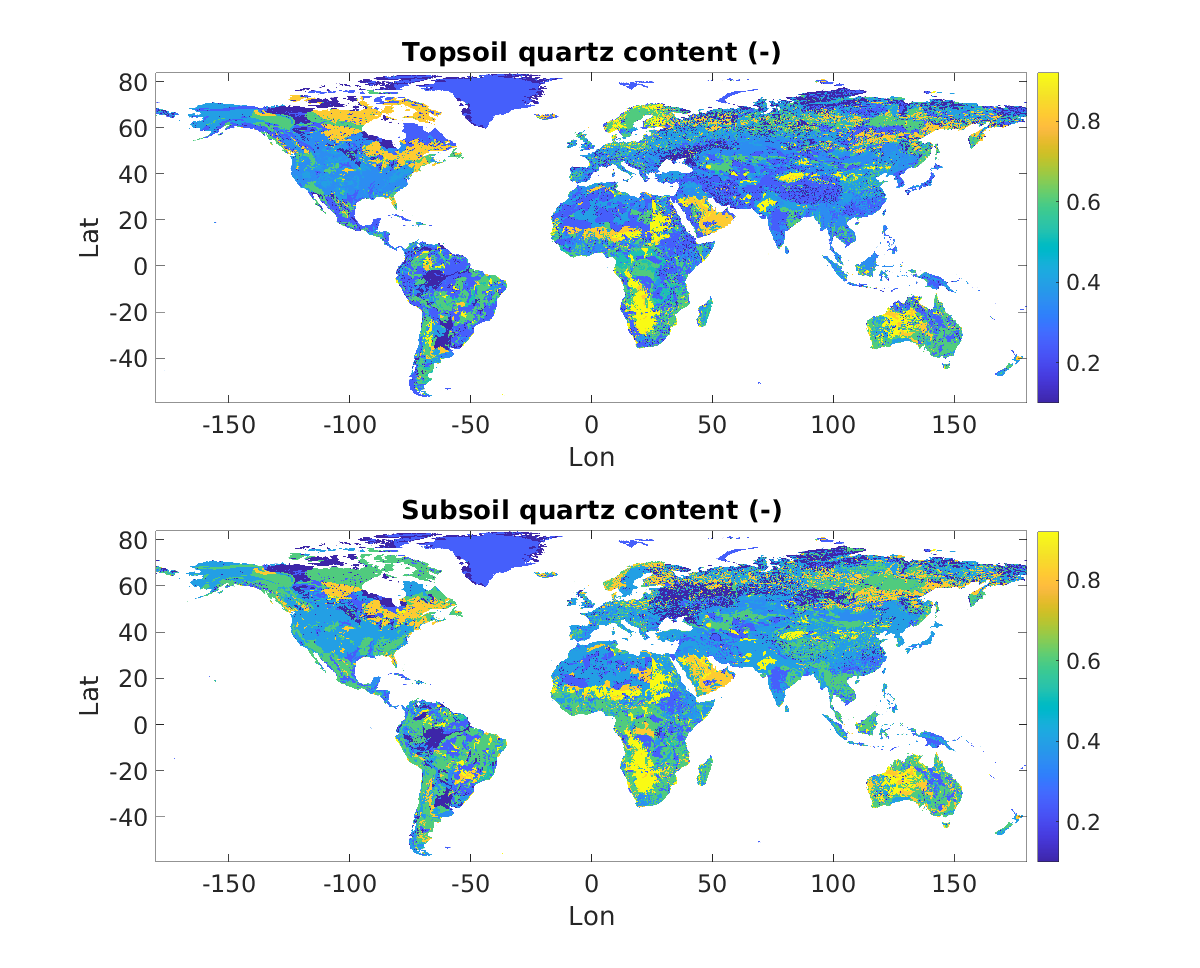


Figure S7. Quartz content, estimated using a pedotransfer table and HWSD soil texture data.


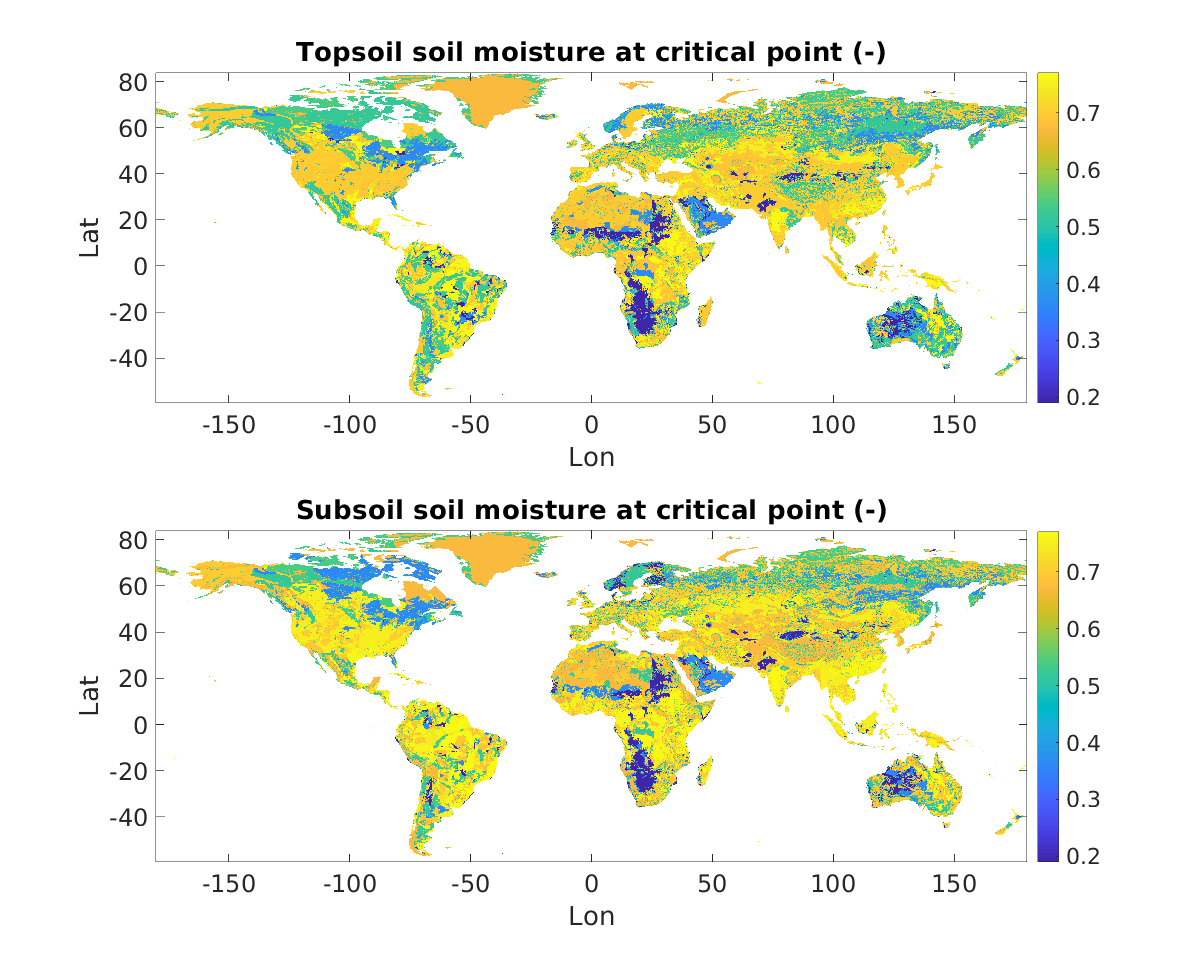


Figure S8. Fractional soil moisture content at the critical point (about 70% of field capacity) *wcr_fract_*.


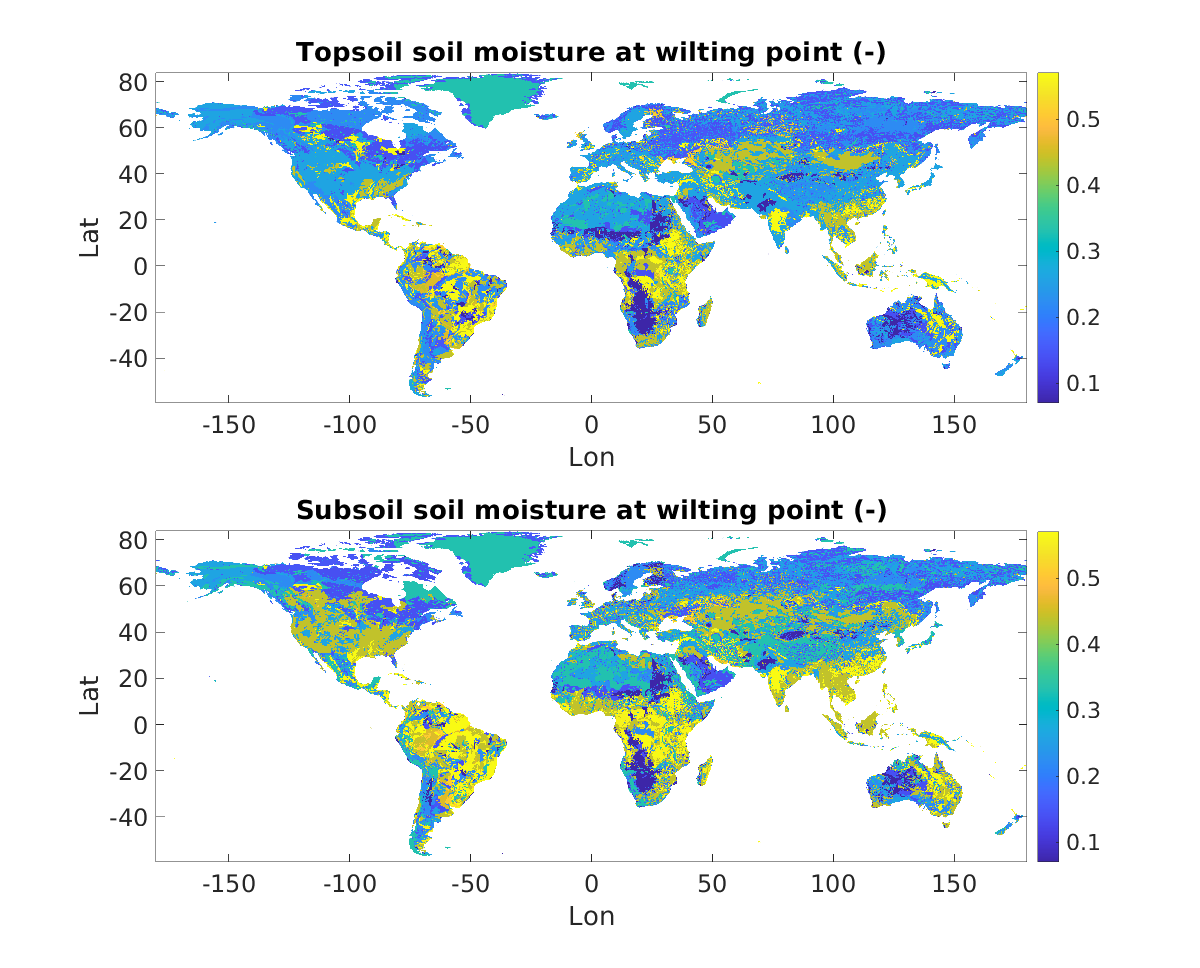


Figure S9. Fractional soil moisture at the wilting point *wpwp_fract_*.


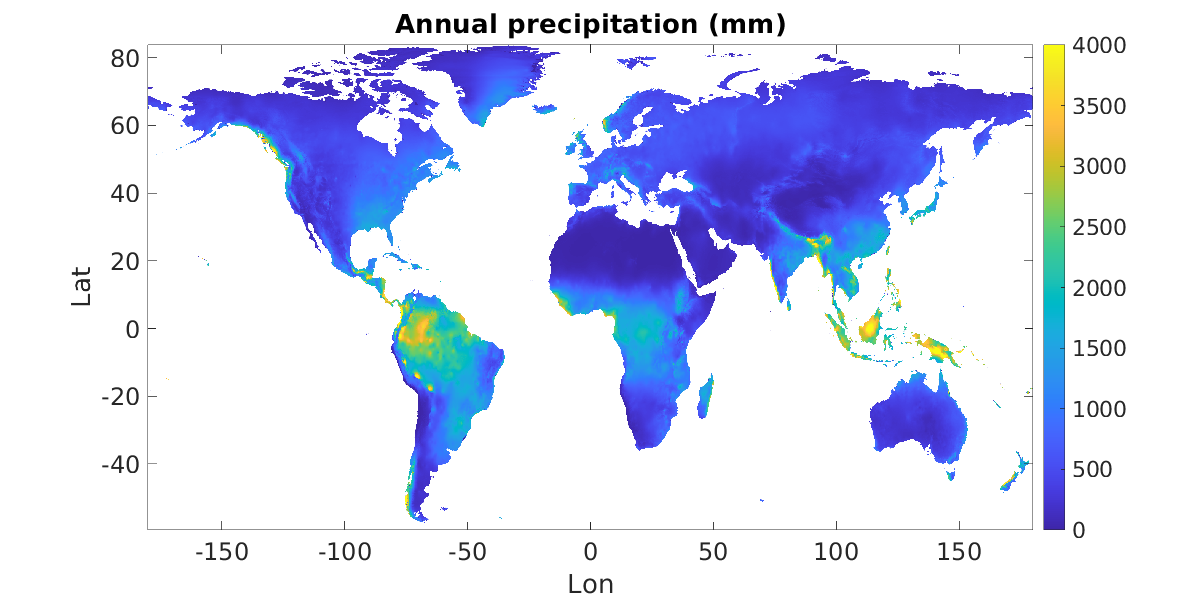


Figure S10. Average annual precipitation from WorldClim. Values greater than 4000 mm have been censored to show spatial variability.


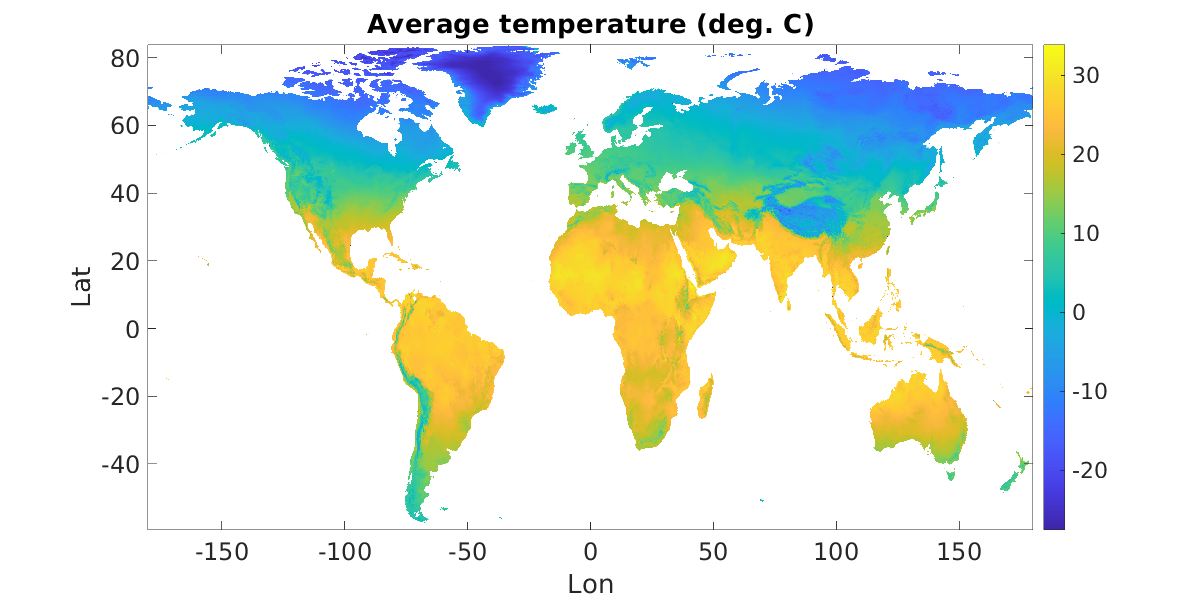


Figure S11. Average temperature from WorldClim, used as the bottom boundary condition for the VIC model’s soil heat flux algorithms.


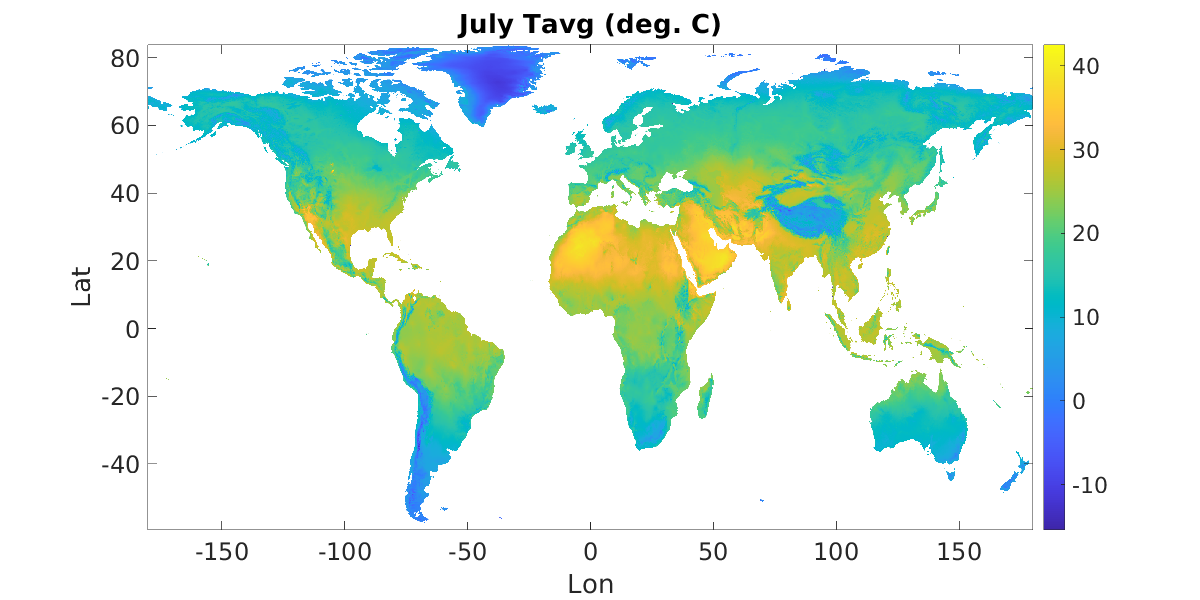


Figure S12. Average July air temperature, used for treeline computations if this option is chosen in the VIC global parameter file.


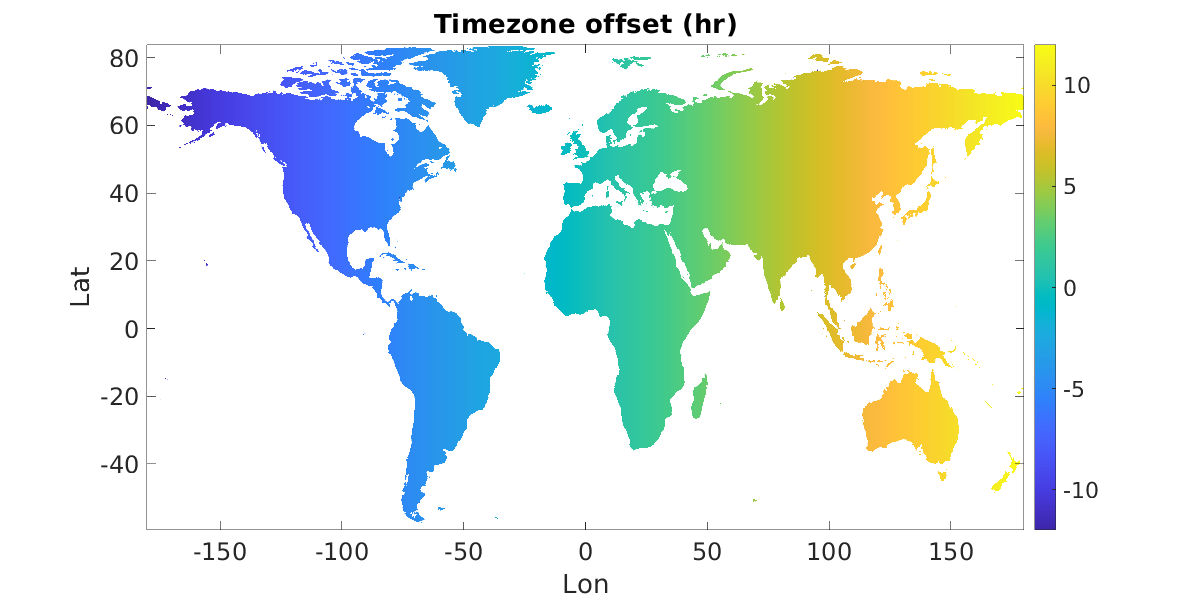


Figure S13. Offgmt, the time zone offset from Greenwich Mean Time/UTC.


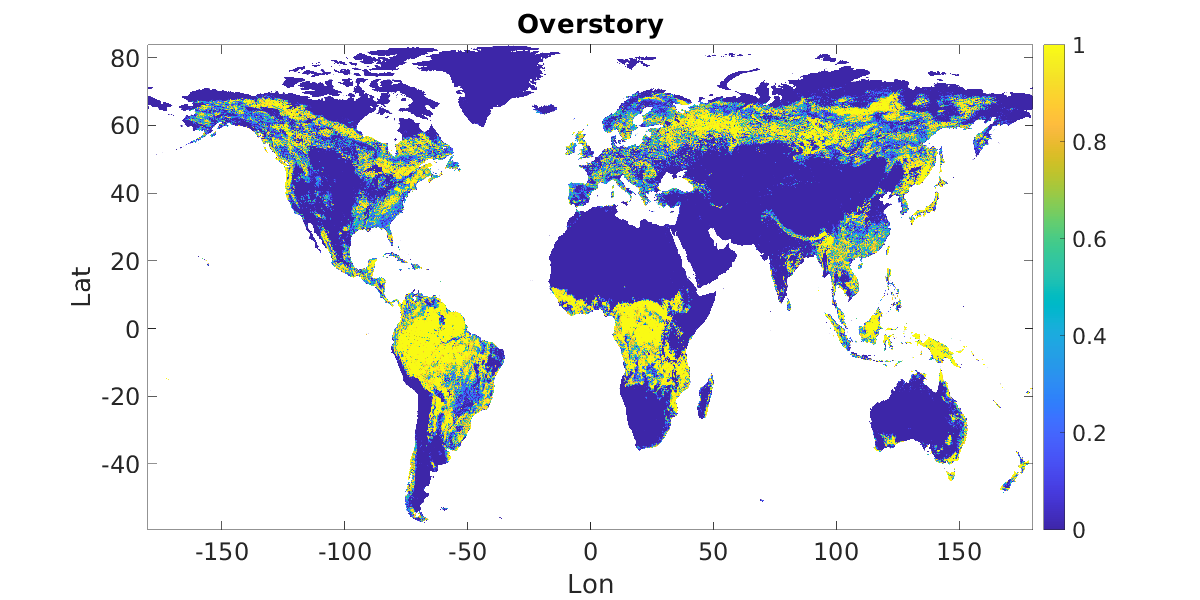


Figure S14. This map shows the area-weighted overstory fraction averaged over all the land cover classes within a single 1/16° grid cell. Overstory equals one if a vegetation cover class has an overstory and zero otherwise.


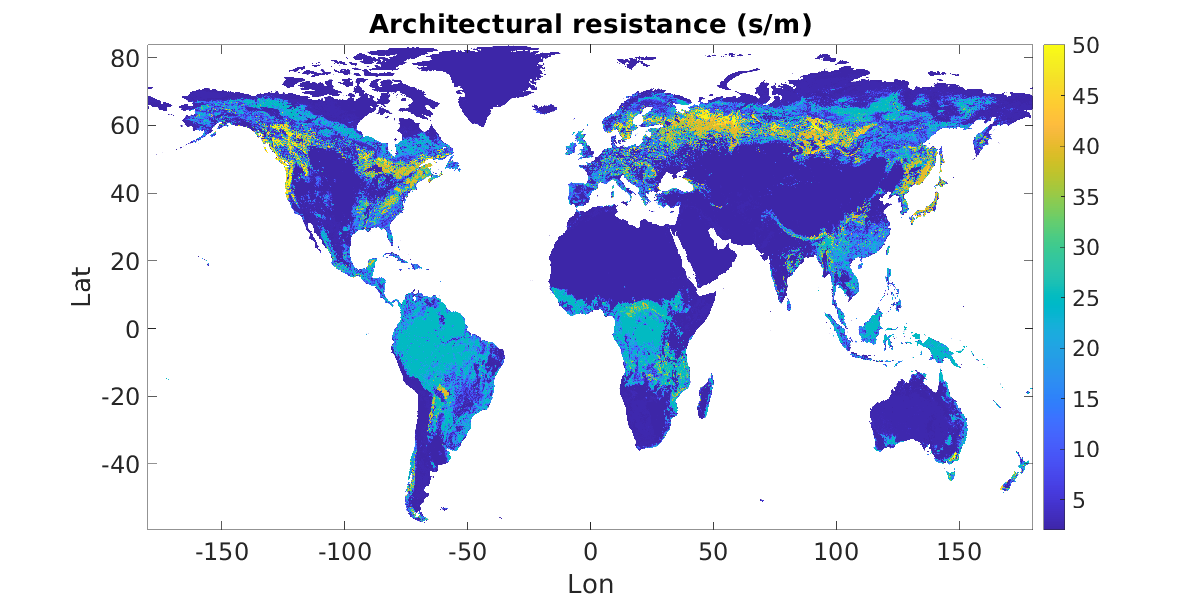


Figure S15. Architectural resistance *r_0_*, shown averaged over the different land cover classes for a given grid cell.


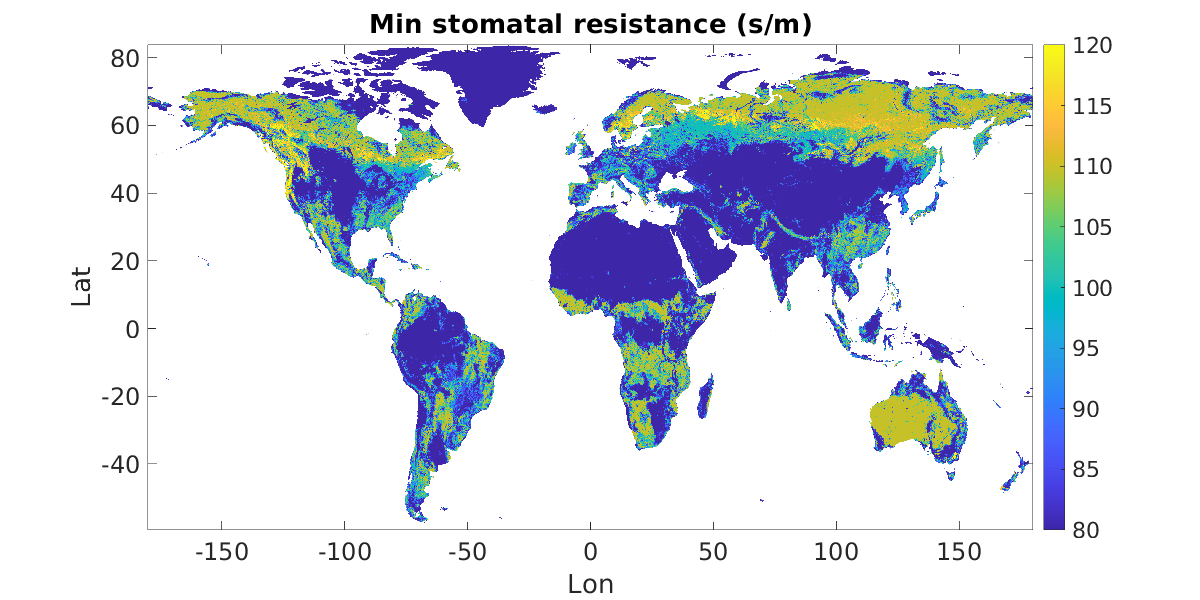


Figure S16. Minimum stomatal resistance *r_min_* for a land cover class, shown averaged over the different land cover classes for a given grid cell.


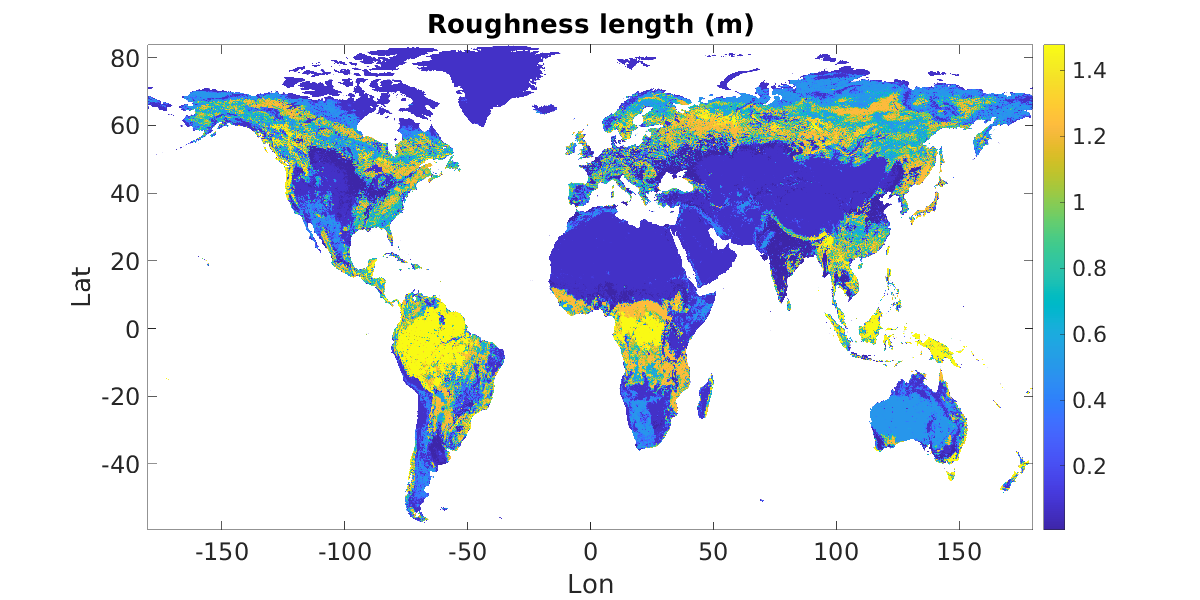


Figure S17. Vegetation roughness length, shown averaged over the different land cover classes for a given grid cell.


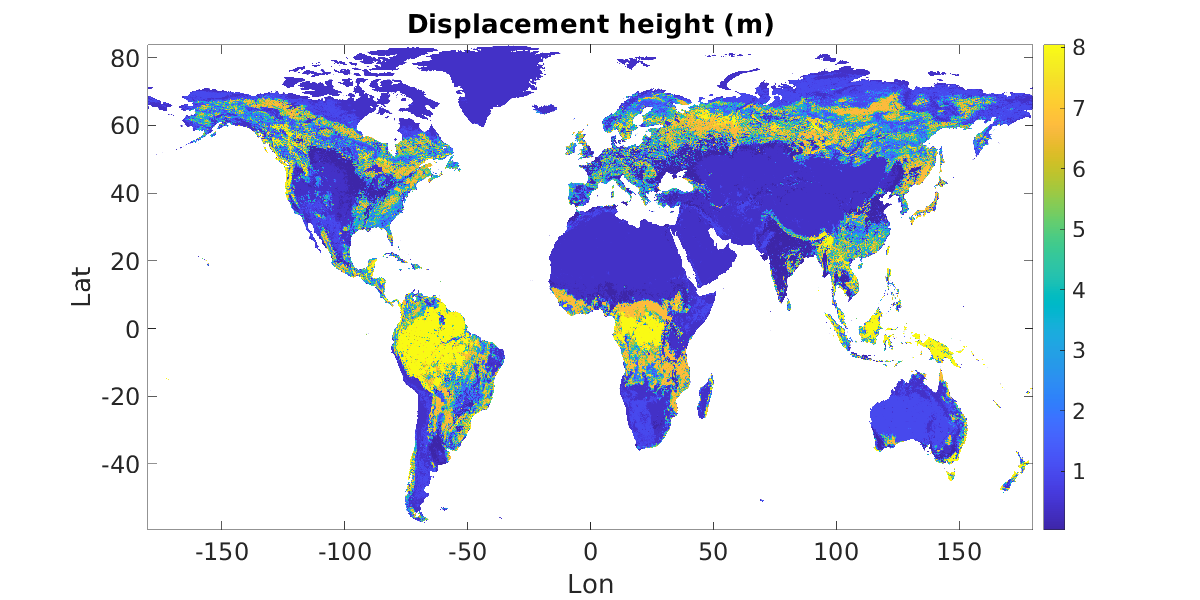


Figure S18. Vegetation displacement height, shown averaged over the different land cover classes for a given grid cell.


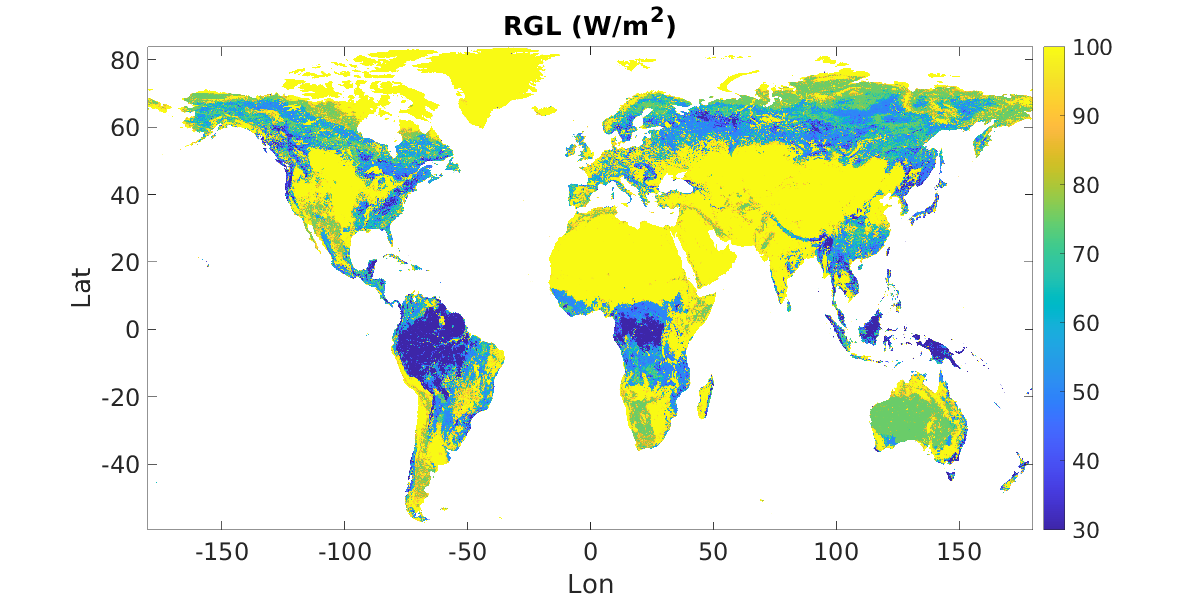


Figure S19. Minimum incoming shortwave radiation in order for transpiration to occur (RGL), shown averaged over the different land cover classes for a given grid cell. Typically, this is about 30 W/m2 for trees and 100 W/m2 for crops, according to the VIC documentation.


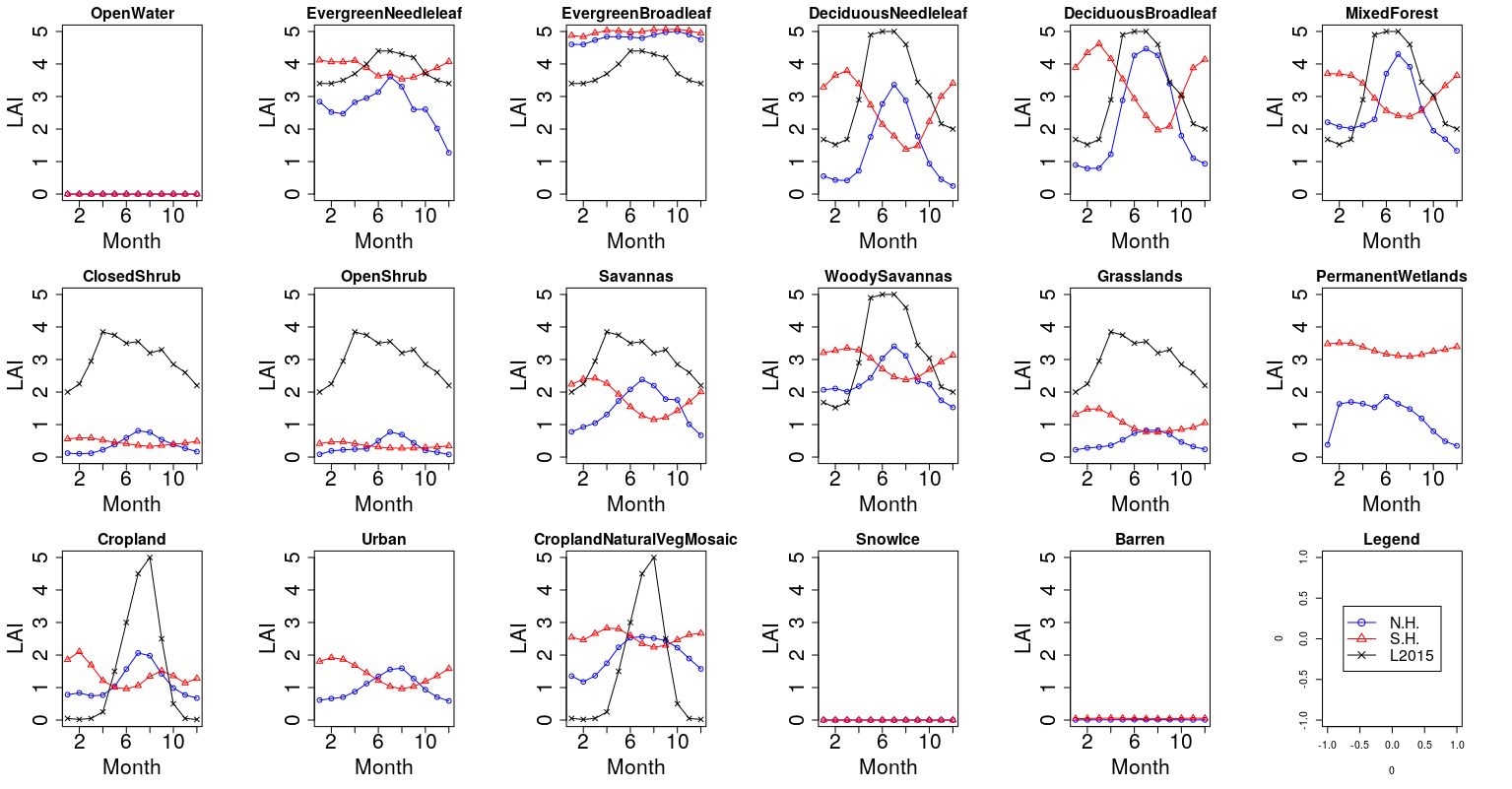


Figure S20. Hemisphere-average monthly leaf-area index (LAI) values for each IGBP land cover type. Gray lines show comparison to L2013 parameter values, when available. LAI values calculated using snow-free MODIS observations from 2017.


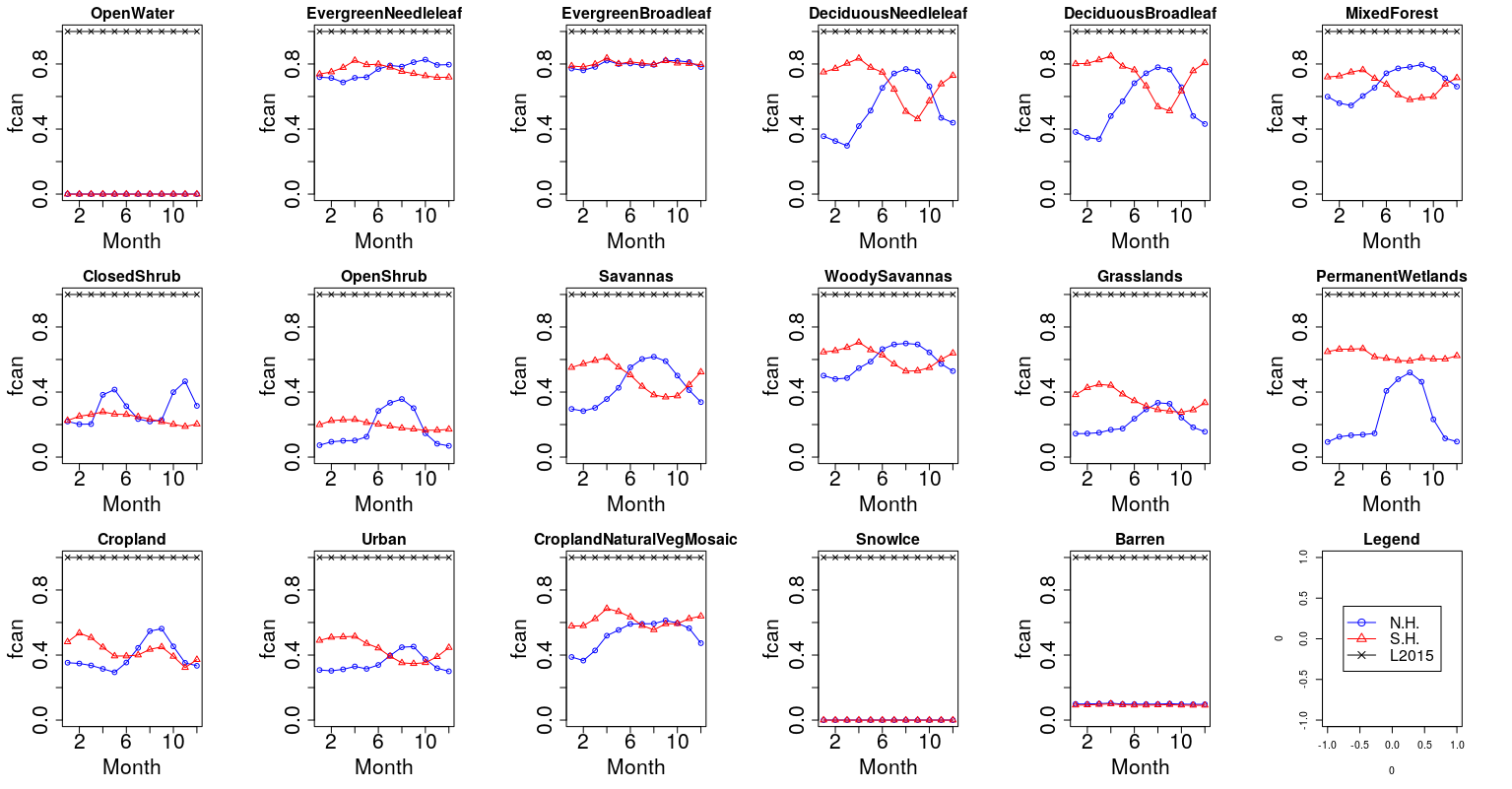


Figure S21. Hemisphere-average monthly canopy cover fraction (fcan) for each IGBP land cover type. Gray lines show comparison to L2013 parameter values, when available. Fcan values calculated using snow-free MODIS observations from 2017.


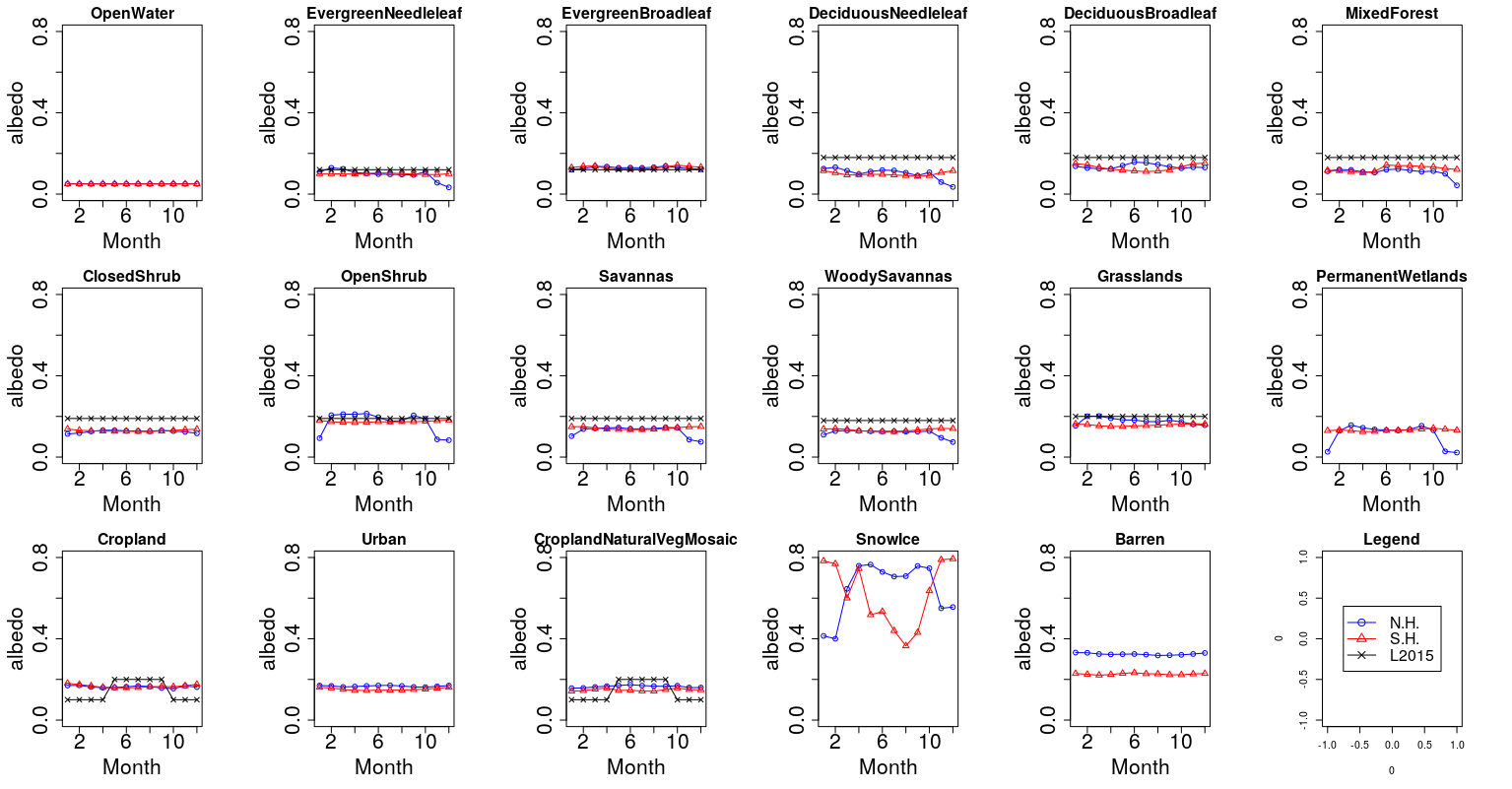


Figure S22. Hemisphere-average monthly albedo values for each IGBP land cover type. Black lines show comparison to L2013 parameter values, when available. Albedo values calculated using snow-free MODIS observations from 2017.
